# Supplementary material for: A systematic comparison of chloroplast genome assembly tools
Source: Genome Biol. 2020 Sep 28;21:254. doi: 10.1186/s13059-020-02153-6 (PMC7520963; doi:10.1186/s13059-020-02153-6)
Supplement: Supplementary file 1 — Additional file 1 Supplemental data. Supplementary data contain a complete list of all real data sets used in this study. Additionally, a table with more details on the used docker images and the detailed results of the performance measurement are included. The file is available at [83]. [file 13059_2020_2153_MOESM1_ESM.pdf]

A systematic comparison of chloroplast genome assembly tools

Supplemental material

Jan A Freudenthal, Simon Pfaff, Niklas Terhoeven, Arthur Korte, Markus J Ankenbrand and Frank Förster

List of Tables

S1 Sequencing Read Archive and Genbank accessions for novel chloroplast genomes 2
S2 Summary real data set 3
S3 Docker images used in our benchmark setup. 17
S4 Performance metrics input size 25K 18
S5 Performance metrics input size 250K 19
S6 Performance metrics input size 2.5M 20
S7 Novel data set 21

Table S1: Sequencing Read Archive and Genbank accessions for novel chloroplast genomes

Species	SRA-Accession	Assembler	Uses	Genbank-Accession
Antirrhinum majus subsp. *Striatum*	ERR2744402	`GetOrganelle`	ornamental plant	-
Antirrhinum majus var. *pseudomajus*	ERR2744401	`GetOrganelle`	ornamental plant	-
Arachis magna	SRR8481359	`GetOrganelle`	-	-
Boechera stricta	SRR6821984	`NOVOPlasty`	-	-
Buddleja globosa	SRR6331521	`GetOrganelle`	folk medicine	-
Congea tomentosa	SRR6940055	`GetOrganelle`	-	-
Cucurbita argyrosperma	SRR2531270	`GetOrganelle`	crop plant	-
Cucurbita ecuadorensis	SRR2531278	`GetOrganelle`	-	-
Cucurbita lundelliana	SRR2531284	`GetOrganelle`	-	-
Cucurbita okeechobeensis	SRR2531280	`GetOrganelle`	-	-
Cucurbita pedatifolia	SRR2531289	`GetOrganelle`	-	-
Cuscuta campestris	ERR1917165	`GetOrganelle`	-	-
Erysimum baeticum	SRR7223704	`GetOrganelle`	-	-
Erysimum nevadense	SRR7223710	`GetOrganelle`	-	-
Garrya flavescens	SRR2401791	`GetOrganelle`	-	-
Gerbera hybrid cultivar	SRR2154064	`GetOrganelle`	-	-
Leucanthemum vulgare	ERR3089127	`GetOrganelle`	ornamental plant	-
Petraeovitex bambusetorum	SRR6940081	`GetOrganelle`	-	-
Pityopsis graminifolia var. *Graminifolia*	SRR4457826	`GetOrganelle`	ornamental plant	-
Pityopsis graminifolia var. *tenuifolia*	SRR4457835	`GetOrganelle`	ornamental plant	-

Table S2: **Summary real data set.** List of real data sets used during our benchmark study. The tables was sorted by species name. Additionally, the table contains the accession for the Sequencing Read Archive (SRA Acc) and the accession of the used reference (Ref Acc). Moreover, the original number of read pairs (read pairs), the overall number of sequenced nucleotides (base pairs), and the mean read length (read len.) is given. Finally, the last column contains the information, if that data set was used for the check of the consistency.

Taxon	SRA Acc	Ref Acc	read pairs	base pairs (bp)	read len. (bp)	Consistency check
Abelmoschus esculentus	SRR5812498	NC_035234	9 028 889	2 726 724 478	151	Yes
Abies sibirica	ERR268415	NC_035067	157 369 451	31 473 890 200	100	NO
Actinidia chinensis	DRR083750	NC_026690	1 974 014	574 124 022	145	Yes
Aegilops sharonensis	ERR359708	NC_024816	179 478 974	36 254 752 748	101	NO
Aegilops speltoides	ERR424849	NC_022135	173 225 639	34 991 579 078	101	NO
Aegilops tauschii	SRR091633	NC_022133	114 178 484	23 064 053 768	101	NO
Ajuga reptans	SRR6940062	NC_023102	16 864 855	5 059 456 500	150	NO
Aldrovanda vesiculosa	SRR7072768	NC_035416	3 510 392	1 060 138 384	151	Yes
Allium cepa	SRR1686960	NC_024813	90 042 453	27 099 552 373	150	Yes
Allium sativum	SRR5602598	NC_031829	1 270 060	748 290 736	295	NO
Alloteropsis angusta	SRR7528995	NC_027951	18 695 483	9 347 741 500	250	NO
Alloteropsis cimicina	SRR7529015	NC_027952	118 496 918	59 248 459 000	250	Yes
Alloteropsis paniculata	SRR4051980	NC_032078	4 048 486	796 542 955	98	NO
Alloteropsis semialata	SRR7528994	NC_027824	26 426 678	13 213 339 000	250	Yes
Aloysia citrodora	SRR5602597	NC_034695	1 423 723	844 386 488	297	NO
Althaea officinalis	SRR5602596	NC_034701	1 409 711	833 584 333	296	NO
Amaranthus hypochondriacus	SRR3399331	NC_030770	6509	3 113 188	239	NO
Ammopiptanthus nanus	SRR6175451	NC_034743	189 485 569	56 845 670 700	150	NO
Anacardium occidentale	SRR5812497	NC_035235	27 997 137	7 055 278 524	126	NO
Apostasia odorata	SRR6037860	NC_030722	48 546 649	14 563 994 700	150	Yes
Arabidopsis arenicola	DRR054584	NC_030346	31 543 276	6 371 741 752	101	Yes
Arabidopsis arenosa	SRR4128972	NC_029334	129 638 900	38 891 670 000	150	Yes

Continued on next page

Table S2 – *Continued from previous page*

Taxon	SRA Acc	Ref Acc	read pairs	base pairs (bp)	read len. (bp)	Consistency check
Arabidopsis cebennensis	SRR2040775	NC_029335	20 358 302	4 112 377 004	101	Yes
Arabidopsis croatica	SRR7637319	NC_030347	14 265 071	3 573 463 604	125	NO
Arabidopsis halleri	SRR8040824	NC_034366	128 303 531	38 747 666 362	151	Yes
Arabidopsis lyrata subsp. lyrata	SRR1705621	NC_034379	16 771 948	4 796 683 328	143	NO
Arabidopsis lyrata	SRR8157563	NC_034365	46 678 588	9 429 074 776	101	NO
Arabidopsis neglecta	SRR2040831	NC_030348	52 410 983	10 482 196 600	100	Yes
Arabidopsis pedemontana	SRR2040802	NC_029336	28 098 774	5 675 952 348	101	NO
Arabidopsis petrogena	SRR2040807	NC_030349	32 189 424	6 502 263 648	101	Yes
Arabidopsis suecica	SRR2084154	NC_030350	158 377 327	31 675 465 400	100	NO
Arabidopsis thaliana	SRR3340908	NC_000932	6 829 274	4 097 564 400	300	NO
Arabidopsis umezawana	SRR2040810	NC_030351	24 335 095	4 915 689 190	101	NO
Arabis alpina	SRR7880736	NC_023367	12 603 925	3 126 123 117	124	Yes
Artemisia annua	SRR5602595	NC_034683	665 200	330 541 594	248	Yes
Arundo plinii	SRR4319202	NC_034652	10 180 635	6 099 219 728	300	Yes
Asclepias nivea	SRR934044	NC_022431	1 637 178	311 063 820	95	NO
Asclepias syriaca	SRR934060	NC_022432	46 704 483	9 434 305 566	101	NO
Asparagus officinalis	DRR056675	NC_034777	83 276 068	16 821 765 736	101	Yes
Astragalus mongholicus	SRR3938254	NC_029828	7 500 181	1 514 903 465	101	Yes
Avena sativa	SRR6056489	NC_027468	115 104 823	57 552 411 500	250	NO
Averrhoa carambola	ERR2799539	NC_033350	1 023 284	309 031 768	151	NO
Betula nana	ERR2026268	NC_033978	45 426 355	13 797 661 576	152	NO
Boswellia sacra	SRR5602594	NC_029420	2 012 947	1 199 195 198	298	Yes
Botryococcus braunii	SRR3721649	NC_025545	137 313 108	41 193 932 400	150	NO
Brachypodium distachyon	SRR4159530	NC_011032	51 410 001	15 525 820 302	151	Yes
Brassica juncea	SRR2057910	NC_028272	54 890 460	11 087 872 920	101	NO
Brassica napus	SRR6856349	NC_016734	34 913 922	10 544 004 444	151	NO
Brassica nigra	SRR2054762	NC_030450	57 198 732	11 554 143 864	101	Yes

Continued on next page

Table S2 – *Continued from previous page*

Taxon	SRA Acc	Ref Acc	read pairs	base pairs (bp)	read len. (bp)	Consistency check
Bupleurum latissimum	SRR6048019	NC_033346	5 702 505	3 432 908 010	301	Yes
Cajanus cajan	SRR073338	NC_031429	132 406 303	26 746 073 206	101	Yes
Camelina sativa	SRR2082664	NC_029337	10 643 512	2 149 989 424	101	Yes
Camellia sinensis	SRR5894861	NC_020019	4 975 058	2 985 034 800	300	NO
Cannabis sativa	SRR7285294	NC_026562	162 968 810	42 761 008 291	131	Yes
Capsella bursa-pastoris	SRR5412136	NC_009270	15 148 015	9 391 769 300	310	NO
Capsella grandiflora	SRR1508428	NC_028517	98 396 864	19 679 372 800	100	NO
Capsella rubella	ERR636124	NC_027693	40 236 079	12 151 295 858	151	NO
Capsicum annuum	SRR1982881	NC_018552	10 804 729	1 966 460 678	91	Yes
Carica papaya	DRR019500	NC_010323	103 259 953	22 510 669 754	109	Yes
Carnegiea gigantea	SRR5036293	NC_027618	12 642 777	7 585 666 200	300	NO
Carthamus tinctorius	SRR2154065	NC_030783	9 648 870	1 949 071 740	101	NO
Castanea mollissima	SRR8731963	NC_014674	118 123 542	35 437 062 600	150	Yes
Catharanthus roseus	SRR5217671	NC_021423	23 799 598	7 139 879 400	150	Yes
Cenchrus americanus	SRR5204424	NC_024171	149 257 429	36 289 690 596	122	Yes
Centaurea diffusa	SRR2729212	NC_024286	69 630 024	13 926 004 800	100	Yes
Chenopodium quinoa	SRR5938314	NC_034949	41 331 617	20 748 471 734	251	NO
Chlamydomonas reinhardtii	SRR4235199	NC_005353	4 795 246	1 448 164 292	151	Yes
Chlorella sorokiniana	SRR4292644	NC_023835	24 148 422	4 877 981 244	101	NO
Chlorella variabilis	SRR7012756	NC_015359	16 059 428	4 837 652 469	151	NO
Chromochloris zofingiensis	SRR5310953	NC_029672	63 205 006	12 641 001 200	100	Yes
Chrysobalanus icaco	SRR1179655	NC_024061	5 544 469	1 119 982 738	101	Yes
Cicer arietinum	SRR5183095	NC_011163	37 577 869	6 764 016 420	90	Yes
Cinnamomum verum	SRR5812493	NC_035236	4 931 460	1 489 300 920	151	NO
Citrullus lanatus	SRR8523014	NC_032008	27 574 511	16 544 706 600	300	Yes
Citrus aurantiifolia	SRR6188466	NC_024929	132 426 000	26 485 200 000	100	NO
Citrus limon	SRR5602593	NC_034690	1 362 792	666 398 607	244	NO

Continued on next page

Table S2 – *Continued from previous page*

Taxon	SRA Acc	Ref Acc	read pairs	base pairs (bp)	read len. (bp)	Consistency check
Citrus maxima	SRR3407098	NC_034290	149 418 786	47 814 011 520	160	NO
Citrus reticulata	SRR3747540	NC_034671	53 225 257	15 967 577 100	150	Yes
Citrus sinensis	SRR3927459	NC_008334	46 781 458	14 034 437 400	150	NO
Coffea arabica	SRR5602572	NC_008535	2 532 608	1 494 738 062	295	NO
Coffea canephora	ERR321701	NC_030053	33 362 762	7 206 356 592	108	Yes
Corymbia torelliana	SRR4063804	NC_028410	66 804 809	20 041 442 700	150	Yes
Couepia guianensis	SRR1179648	NC_024063	7 411 159	1 497 054 118	101	Yes
Cucumis melo subsp. melo	ERR246536	NC_015983	33 207 205	10 094 990 320	152	NO
Cucumis sativus	SRR6490504	NC_007144	35 348 114	10 646 772 194	151	Yes
Cunninghamia lanceolata	ERR2799542	NC_021437	1 062 614	320 909 428	151	Yes
Cymbidium ensifolium	SRR6117755	NC_028525	12 766 736	7 685 575 072	301	Yes
Cymbidium kanran	SRR6117756	NC_029711	5 534 224	3 331 602 848	301	NO
Cymbidium lancifolium	SRR6117757	NC_029712	5 856 776	3 525 779 152	301	NO
Cymbidium macrorhizon	SRR6117754	NC_029713	5 777 841	3 478 260 282	301	NO
Cynodon dactylon	SRR5457035	NC_034680	121 672	65 496 192	269	Yes
Cytinus hypocistis	ERR964904	NC_031150	8 664 415	1 750 211 830	101	NO
Dactylis glomerata	SRR5236602	NC_027473	131 404 213	39 421 263 900	150	NO
Daucus carota	SRR7601255	NC_008325	208 788 541	63 054 139 382	151	Yes
Dendrobium aphyllum	SRR3932123	NC_035322	24 390 720	7 317 216 000	150	Yes
Dendrobium chrysanthum	SRR3932147	NC_035336	12 371 613	3 711 483 900	150	Yes
Dendrobium chrysotoxum	SRR3932141	NC_028549	12 969 805	3 890 941 500	150	Yes
Dendrobium crepidatum	SRR3932124	NC_035331	10 220 355	3 066 106 500	150	NO
Dendrobium denneanum	SRR3932144	NC_035324	11 765 400	3 529 620 000	150	Yes
Dendrobium falconeri	SRR3932139	NC_035326	11 326 112	3 397 833 600	150	NO
Dendrobium nobile	SRR3932122	NC_029456	11 410 037	3 423 011 100	150	Yes
Dendrobium parishii	SRR3932128	NC_035339	14 183 700	4 255 110 000	150	NO
Dendrobium pendulum	SRR3932138	NC_029705	11 357 541	3 407 262 300	150	Yes

Continued on next page

Table S2 – *Continued from previous page*

Taxon	SRA Acc	Ref Acc	read pairs	base pairs (bp)	read len. (bp)	Consistency check
Dendrobium primulinum	SRR3932120	NC_035321	10 864 205	3 259 261 500	150	NO
Dendrobium wardianum	SRR3932135	NC_035329	9 367 500	2 810 250 000	150	Yes
Deschampsia antarctica	SRR1158316	NC_023533	153 346 825	30 976 058 650	101	Yes
Digitalis lanata	SRR5602573	NC_034688	1 257 524	730 269 772	290	NO
Dionaea muscipula	SRR7072324	NC_035417	16 215 933	4 897 211 766	151	NO
Dioscorea rotundata	DRR063110	NC_024170	18 637 125	9 295 053 322	249	NO
Dioscorea villosa	SRR5602590	NC_034686	1 447 023	858 275 557	297	Yes
Diospyros lotus	SRR1560932	NC_030786	8 240 803	1 648 160 600	100	Yes
Drosera erythrorhiza	SRR7072766	NC_035241	5 832 398	1 761 384 196	151	NO
Drosera regia	SRR7072322	NC_035415	1 341 304	405 073 808	151	NO
Dunaliella salina	SRR3923695	NC_016732	164 049 728	49 214 918 400	150	Yes
Echinacea angustifolia	SRR5602579	NC_034324	1 669 371	878 253 310	263	Yes
Echinacea atrorubens	SRR5602578	NC_034323	961 923	472 814 810	246	NO
Echinacea laevigata	SRR5602581	NC_034322	1 099 311	545 173 423	248	NO
Echinacea pallida	SRR5602580	NC_034321	2 039 307	832 368 111	204	Yes
Echinacea paradoxa	SRR5602575	NC_034320	3 101 240	1 692 835 034	273	NO
Echinacea purpurea	SRR5602574	NC_034327	5 197 414	2 531 457 606	244	Yes
Echinacea sanguinea	SRR5602577	NC_034328	4 911 880	2 224 965 457	226	Yes
Echinacea speciosa	SRR5602576	NC_034325	970 715	483 247 492	249	NO
Echinacea tennesseensis	SRR5602587	NC_034326	907 178	434 759 190	240	NO
Echinochloa crus-galli	SRR5920285	NC_028719	203 467 365	50 866 841 250	125	NO
Echinochloa oryzicola	SRR5902661	NC_024643	129 112 810	32 278 202 500	125	Yes
Elaeis guineensis	ERR276848	NC_017602	143 340 222	28 954 724 844	101	Yes
Eleutherococcus senticosus	SRR5602586	NC_016430	424 930	211 982 751	249	NO
Epimedium koreanum	SRR8534306	NC_029943	8 308 432	1 262 881 664	76	NO
Epimedium sagittatum	SRR8275049	NC_029428	4 141 262	629 471 824	76	NO
Eragrostis tef	SRR1463402	NC_029413	85 023 843	14 454 053 310	85	NO

Continued on next page

Table S2 – *Continued from previous page*

Taxon	SRA Acc	Ref Acc	read pairs	base pairs (bp)	read len. (bp)	Consistency check
Eriobotrya japonica	SRR5602604	NC_034639	1 852 832	918 799 928	248	Yes
Erodium chrysanthum	SRR576525	NC_027065	31 570 835	6 314 167 000	100	NO
Erodium texanum	ERR2799526	NC_014569	1 158 815	349 962 130	151	Yes
Erythranthe lutea	SRR5307620	NC_030212	10 000 000	2 000 000 000	100	Yes
Eucalyptus grandis	SRR1392700	NC_014570	327 608 152	98 282 445 600	150	Yes
Euphorbia esula	SRR6355713	NC_033910	65 533 012	13 106 602 400	100	NO
Fagopyrum tataricum	SRR5433722	NC_027161	15 222 050	6 981 058 500	229	Yes
Festuca arundinacea	SRR6885354	NC_011713	6 197 329	3 085 916 062	249	NO
Ficus carica	SRR5678803	NC_035237	26 827 466	15 073 339 221	281	NO
Ficus racemosa	SRR1405699	NC_028185	31 025 867	4 715 931 784	76	NO
Foeniculum vulgare	SRR8690417	NC_029469	3 715 298	750 490 196	101	NO
Fragaria chiloensis	SRR1612828	NC_019601	43 583 617	10 460 068 080	120	NO
Fragaria vesca subsp. bracteata	SRR866156	NC_018766	4 311 362	819 158 780	95	Yes
Fragaria virginiana	SRR5602605	NC_019602	1 199 457	708 866 398	295	Yes
Francoa sonchifolia	SRR576529	NC_021101	32 806 903	6 561 380 600	100	NO
Geranium maderense	ERR2799530	NC_029999	1 031 538	311 524 476	151	Yes
Ginkgo biloba	SRR3089810	NC_016986	10 164 078	2 541 019 500	125	NO
Glycine dolichocarpa	SRR1174380	NC_021648	44 850 853	9 059 872 306	101	NO
Glycine max	SRR6796784	NC_007942	70 339 371	21 242 490 042	151	NO
Glycine soja	SRR7725010	NC_022868	131 827 844	39 548 353 200	150	Yes
Glycine syndetika	SRR1176843	NC_021650	51 528 078	10 408 671 756	101	NO
Glycine tomentella	SRR6823488	NC_021636	265 989 287	131 814 519 905	248	NO
Glycyrrhiza glabra	SRR8690419	NC_024038	4 940 810	998 043 620	101	Yes
Glyptostrobus pensilis	ERR2799543	NC_031354	1 061 851	320 679 002	151	NO
Gnetum gnemon	ERR268420	NC_026301	146 146 748	29 229 349 600	100	NO
Gossypium anomalum	SRR3560153	NC_023213	152 611 035	30 827 429 070	101	NO
Gossypium arboreum	SRR2012734	NC_016712	8 946 554	5 385 825 508	301	NO

Continued on next page

Table S2 – *Continued from previous page*

Taxon	SRA Acc	Ref Acc	read pairs	base pairs (bp)	read len. (bp)	Consistency check
Gossypium aridum	SRR8136263	NC_033396	158 194 517	31 638 903 400	100	Yes
Gossypium armourianum	SRR8136258	NC_033400	155 005 858	31 001 171 600	100	NO
Gossypium barbadense	SRR8627357	NC_008641	159 103 409	79 869 911 318	251	NO
Gossypium bickii	SRR3560187	NC_023214	179 898 006	35 979 601 200	100	Yes
Gossypium darwinii	SRR8545538	NC_016670	440 216 228	132 945 300 856	151	NO
Gossypium davidsonii	SRR6334584	NC_033395	117 719 642	35 315 892 600	150	NO
Gossypium gossypioides	SRR3560148	NC_017894	153 288 210	30 657 642 000	100	NO
Gossypium harknessii	SRR8136257	NC_033333	183 115 691	36 431 624 874	99	NO
Gossypium herbaceum	SRR2012721	NC_023215	12 440 256	7 489 034 112	301	NO
Gossypium hirsutum	SRR3560152	NC_007944	4 487 735	2 205 113 705	246	Yes
Gossypium klotzschianum	SRR8136264	NC_033394	161 773 353	32 354 670 600	100	NO
Gossypium longicalyx	SRR617704	NC_023216	534 258 839	107 920 285 478	101	Yes
Gossypium mustelinum	SRR8727328	NC_016711	158 115 596	79 374 029 192	251	NO
Gossypium raimondii	ERR1449077	NC_016668	21 720 967	6 559 732 034	151	NO
Gossypium somalense	SRR3560160	NC_018110	69 625 198	13 925 039 600	100	Yes
Gossypium sturtianum	SRR3560184	NC_023218	95 466 009	19 093 201 800	100	NO
Gossypium thurberi	SRR8076131	NC_015204	181 003 659	36 200 731 800	100	Yes
Gossypium tomentosum	SRR6334536	NC_016690	138 849 836	41 654 950 800	150	NO
Gossypium trilobum	SRR3560157	NC_033397	52 816 676	10 563 335 200	100	NO
Gossypium turneri	SRR8136254	NC_026835	124 131 681	37 239 504 300	150	NO
Haberlea rhodopensis	SRR4428742	NC_031852	8 365 536	1 673 107 200	100	NO
Haplostachys haplostachya	SRR3170741	NC_029819	251 836	76 054 472	151	NO
Helianthus annuus	SRR825830	NC_007977	57 562 191	13 124 179 548	114	Yes
Helianthus argophyllus	SRR2155086	NC_030275	12 784 471	2 582 463 142	101	NO
Helianthus debilis	SRR5907791	NC_030173	7 323 836	1 464 767 200	100	NO
Helianthus divaricatus	SRR3492253	NC_023109	13 246 221	2 649 244 200	100	NO
Helianthus giganteus	SRR5907716	NC_023107	20 626 828	4 125 365 600	100	NO

Continued on next page

Table S2 – *Continued from previous page*

Taxon	SRA Acc	Ref Acc	read pairs	base pairs (bp)	read len. (bp)	Consistency check
Helianthus grosseserratus	SRR5907715	NC_023108	23 480 167	4 696 033 400	100	Yes
Heteropogon triticeus	SRR5446059	NC_035025	14 302 809	4 290 842 700	150	Yes
Hibiscus syriacus	SRR1265942	NC_026909	25 909 841	5 233 787 882	101	NO
Hirtella physophora	SRR1179646	NC_024066	18 840 660	3 768 132 000	100	Yes
Hirtella racemosa	SRR1179649	NC_024060	8 044 385	1 624 965 770	101	Yes
Hordeum vulgare subsp. vulgare	SRR490932	NC_008590	262 845 212	65 711 303 000	125	NO
Humulus lupulus	SRR8690418	NC_028032	6 541 057	1 321 293 514	101	Yes
Hydrastis canadensis	SRR5602606	NC_034702	1 356 811	671 823 274	248	Yes
Hyoscyamus niger	SRR1508958	NC_024261	21 000 000	4 200 000 000	100	Yes
Hypseocharis bilobata	SRR576531	NC_023260	32 280 852	6 456 170 400	100	NO
Illicium anisatum	SRR5602608	NC_034703	3 195 974	961 644 484	150	NO
Illicium floridanum	SRR5602609	NC_034685	1 929 116	1 141 799 529	296	NO
Illicium henryi	SRR5602610	NC_034699	1 240 196	611 286 832	246	NO
Illicium verum	SRR5602607	NC_034689	2 752 799	828 427 309	150	NO
Ipomoea batatas	SRR7868503	NC_026703	55 433 003	27 827 367 506	251	NO
Ipomoea nil	DRR013917	NC_031159	149 492 354	44 847 706 200	150	NO
Ipomoea trifida	SRR6667669	NC_034670	148 892 802	47 645 696 640	160	NO
Jasminum sambac	SRR5602611	NC_034694	4 487 750	2 661 867 968	297	NO
Jasminum tortuosum	SRR5602601	NC_034691	1 468 745	726 257 599	247	NO
Jatropha curcas	DRR001794	NC_012224	99 881 947	15 182 055 944	76	NO
Juglans regia	SRR2057822	NC_028617	111 991 280	33 821 366 560	151	Yes
Juniperus cedrus	SRR1145775	NC_028190	46 193 335	9 238 667 000	100	Yes
Juniperus communis	ERR268423	NC_035068	472 881 723	94 576 344 600	100	NO
Lactuca sativa	SRR8736654	NC_007578	267 081 494	80 124 448 200	150	Yes
Lathyrus sativus	ERR413118	NC_014063	3 876 258	783 004 116	101	NO
Laurus nobilis	SRR5602602	NC_034700	1 774 932	880 729 409	248	Yes
Lavandula angustifolia	SRR5757713	NC_029370	37 808 338	11 418 118 076	151	NO

Continued on next page

Table S2 – *Continued from previous page*

Taxon	SRA Acc	Ref Acc	read pairs	base pairs (bp)	read len. (bp)	Consistency check
Lens culinaris	ERR413115	NC_027152	6 895 415	1 392 873 830	101	Yes
Licania alba	SRR1179652	NC_024064	6 960 753	1 406 072 106	101	Yes
Licania sprucei	SRR1179651	NC_024065	6 492 846	1 311 554 892	101	NO
Lilium tsingtauense	SRR1265940	NC_027675	9 313 992	1 881 426 384	101	NO
Liriodendron tulipifera	SRR4240953	NC_008326	4 000 000	1 208 000 000	151	Yes
Litchi chinensis	SRR5812499	NC_035238	6 720 070	2 029 461 140	151	NO
Lotus japonicus	SRR8115522	NC_002694	175 495 827	52 999 739 754	151	Yes
Ludisia discolor	SRR3484539	NC_030540	14 073 966	8 472 527 532	301	Yes
Macadamia integrifolia	ERR1397263	NC_025288	28 756 805	8 684 555 110	151	Yes
Magnolia biondii	SRR5602588	NC_034687	1 600 124	953 606 691	298	NO
Magnolia denudata	SRR5602603	NC_018357	1 640 979	978 183 192	298	Yes
Magnolia officinalis subsp. biloba	SRR5602589	NC_020317	1 744 003	1 040 030 660	298	NO
Malus prunifolia	SRR3571175	NC_031163	22 532 714	6 804 879 628	151	NO
Mangifera indica	SRR5812496	NC_035239	9 989 213	3 016 742 326	151	Yes
Manihot esculenta	SRR2847384	NC_010433	121 616 638	54 727 487 100	225	Yes
Mankyua chejuensis	SRR7630500	NC_017006	7 279 904	4 382 502 208	301	Yes
Marchantia polymorpha	SRR396657	NC_001319	123 055 214	36 916 564 200	150	NO
Medicago truncatula	SRR1028837	NC_003119	29 663 436	8 958 357 672	151	NO
Melianthus villosus	SRR576532	NC_023256	31 695 892	6 339 178 400	100	Yes
Mentha longifolia	SRR3204556	NC_032054	67 215 785	13 577 588 570	101	Yes
Miscanthus sacchariflorus	SRR559245	NC_028720	73 073 855	22 068 304 210	151	NO
Miscanthus sinensis	SRR4028761	NC_028721	34 003 586	10 269 082 972	151	Yes
Mitragyna speciosa	SRR5602600	NC_034698	1 327 534	658 729 522	248	NO
Monsonia emarginata	ERR2799532	NC_029694	1 089 971	329 171 242	151	Yes
Nelumbo lutea	SRR5115287	NC_015605	45 729 480	11 432 370 000	125	Yes
Nelumbo nucifera	SRR7159815	NC_025339	30 517 828	9 155 348 400	150	NO
Nicotiana otophora	SRR1171700	NC_032724	67 460 219	13 626 964 238	101	NO

Continued on next page

Table S2 – *Continued from previous page*

Taxon	SRA Acc	Ref Acc	read pairs	base pairs (bp)	read len. (bp)	Consistency check
Nicotiana tabacum	SRR954964	NC_001879	792 406 088	160 066 029 776	101	Yes
Nicotiana undulata	SRR8173251	NC_016068	223 212 812	45 088 988 024	101	Yes
Ocimum basilicum	SRR6940087	NC_035143	32 031 837	9 609 551 100	150	NO
Oenothera biennis	SRR1771524	NC_010361	13 426 811	2 658 508 578	99	Yes
Oenothera villaricae	SRR1771538	NC_030532	3 055 389	611 077 800	100	NO
Olea europaea subsp. europaea	ERR375848	NC_015401	27 590 779	16 729 938 945	303	NO
Oryza australiensis	DRR056778	NC_024608	69 350 090	20 805 027 000	150	NO
Oryza barthii	SRR7341625	NC_027460	55 484 960	27 742 480 000	250	Yes
Oryza brachyantha	DRR053294	NC_030596	10 715 682	3 214 704 600	150	Yes
Oryza glaberrima	SRR7341626	NC_024175	62 571 390	31 285 695 000	250	NO
Oryza glumipatula	DRR057991	NC_027461	23 451 341	7 035 402 300	150	Yes
Oryza longiglumis	DRR056658	NC_034763	50 656 466	15 196 939 800	150	Yes
Oryza longistaminata	DRR058031	NC_027462	17 927 163	5 378 148 900	150	Yes
Oryza meridionalis	DRR058012	NC_016927	19 143 143	5 742 942 900	150	NO
Oryza meyeriana	DRR056670	NC_034765	41 115 373	12 334 611 900	150	NO
Oryza minuta	ERR385916	NC_030298	28 110 596	6 746 543 040	120	Yes
Oryza officinalis	DRR000604	NC_027463	25 866 032	5 690 527 040	110	Yes
Oryza punctata	SRR1264539	NC_027676	8 000 000	1 600 000 000	100	NO
Oryza ridleyi	DRR056663	NC_034764	91 020 902	27 306 270 600	150	Yes
Oryza rufipogon	SRR6220521	NC_017835	26 511 359	8 006 430 418	151	NO
Oryza sativa Indica Group	SRR553489	NC_027678	4 956 305	2 488 065 110	251	NO
Oryza sativa Japonica Group	SRR547959	NC_001320	5 918 441	2 971 057 382	251	NO
Oryza sativa	ERR2696318	NC_031333	6 588 721	3 947 278 012	300	Yes
Ostreococcus tauri	SRR4020477	NC_008289	89 130 001	17 826 000 200	100	NO
Ostrya rehderiana	SRR8302715	NC_028349	33 211 296	8 145 137 640	123	NO
Panax ginseng	SRR5533645	NC_006290	105 595 137	37 246 922 641	176	Yes
Panax japonicus	SRR6512791	NC_028703	71 312 188	21 020 452 500	147	NO

Continued on next page

Table S2 – *Continued from previous page*

Taxon	SRA Acc	Ref Acc	read pairs	base pairs (bp)	read len. (bp)	Consistency check
Panax notoginseng	SRR6512795	NC_026447	7 185 943	2 100 564 900	146	NO
Panax quinquefolius	SRR6512793	NC_027456	20 952 746	6 152 880 600	147	NO
Panax stipuleanatus	SRR6512796	NC_030598	13 610 275	3 922 500 000	144	Yes
Panicum capillare	SRR485871	NC_030493	219 099 842	43 819 968 400	100	Yes
Panicum miliaceum	SRR6650558	NC_029732	159 892 965	80 266 268 430	251	Yes
Panicum virgatum	SRR8440701	NC_015990	92 006 218	46 187 121 436	251	NO
Papaver somniferum	SRR6780757	NC_029434	480 182 430	252 410 803 808	263	NO
Parinari campestris	SRR1179645	NC_024067	5 970 365	1 206 013 730	101	NO
Paulownia tomentosa	SRR6940033	NC_031436	29 457 173	8 837 151 900	150	NO
Pelargonium citronellum	SRR576534	NC_031194	30 750 530	6 150 106 000	100	Yes
Pelargonium cotyledonis	ERR2799534	NC_028052	995 842	300 744 284	151	NO
Pelargonium x hortorum	SRR576536	NC_008454	31 358 909	6 271 781 800	100	NO
Perilla frutescens	SRR6940083	NC_030756	34 758 180	10 427 454 000	150	Yes
Phaseolus vulgaris	SRR5807695	NC_009259	17 399 674	5 289 500 896	152	Yes
Phoenix dactylifera	SRR2518264	NC_013991	89 471 372	16 641 675 192	93	NO
Phyllostachys edulis	SRR8245858	NC_015817	196 487 218	58 946 165 400	150	NO
Physcomitrella patens	ERR1638191	NC_005087	97 932 953	29 379 885 900	150	Yes
Picea abies	ERR1727007	NC_021456	2 556 898	644 338 296	126	NO
Picea asperata	ERR1735493	NC_032367	1 862 931	469 458 612	126	Yes
Picea crassifolia	ERR1735613	NC_032366	1 706 374	430 006 248	126	NO
Picea glauca	SRR869482	NC_028594	19 568 105	19 568 105 000	500	Yes
Picea jezoensis	ERR1795324	NC_029374	2 499 244	629 809 488	126	Yes
Picea sitchensis	SRR5028182	NC_011152	28 752 590	14 376 295 000	250	Yes
Pimenta dioica	SRR5602585	NC_034684	1 821 150	1 068 525 583	293	Yes
Pinus lambertiana	SRR2026990	NC_011156	165 888 035	49 959 743 872	151	Yes
Pinus massoniana	SRR7666034	NC_021439	6 755 123	2 026 536 900	150	NO
Pinus sylvestris	ERR268429	NC_035069	162 706 395	32 541 279 000	100	NO

Continued on next page

Table S2 – *Continued from previous page*

Taxon	SRA Acc	Ref Acc	read pairs	base pairs (bp)	read len. (bp)	Consistency check
Pinus taeda	SRR1049756	NC_021440	4 969 100	2 534 241 000	255	NO
Piper auritum	SRR5602592	NC_034697	1 951 892	964 587 352	247	NO
Piper nigrum	SRR5602591	NC_034692	1 342 936	797 054 024	297	NO
Pistacia vera	SRR4453367	NC_034998	62 397 980	18 719 394 000	150	Yes
Pisum sativum	SRR6702454	NC_014057	144 850 725	29 259 846 450	101	NO
Podococcus barteri	SRR2120220	NC_027276	2453	671 984	137	Yes
Populus alba	SRR3678826	NC_008235	40 869 133	8 255 564 866	101	Yes
Populus tremula x Populus alba	SRR1653109	NC_028504	9 920 342	2 003 909 084	101	NO
Populus tremula	ERR1735633	NC_027425	3 234 148	815 005 296	126	Yes
Populus trichocarpa	SRR5467392	NC_009143	298 329 746	149 761 532 492	251	NO
Premna microphylla	SRR6940036	NC_026291	22 624 524	6 787 357 200	150	Yes
Primula veris	SRR1660449	NC_031428	3 263 725	1 599 834 089	245	NO
Prunus dulcis	SRR5602582	NC_034696	1 285 872	631 355 142	245	NO
Prunus kansuensis	SRR3138168	NC_023956	72 248 792	14 449 758 400	100	Yes
Prunus mume	SRR8240066	NC_023798	45 570 976	13 671 292 800	150	Yes
Prunus persica	SRR5073560	NC_014697	9 978 569	4 989 866 485	250	NO
Psidium guajava	SRR5812495	NC_033355	20 356 683	5 129 884 116	126	NO
Punica granatum	SRR5812494	NC_035240	7 814 299	2 359 918 298	151	NO
Pyrus pyrifolia	SRR5196234	NC_015996	33 563 581	9 782 203 050	146	Yes
Raphanus sativus	DRR014095	NC_024469	513 274 067	103 681 361 534	101	NO
Rhazya stricta	ERR351509	NC_024292	36 920 951	6 645 771 180	90	NO
Saccharum officinarum	SRR7771854	NC_035224	91 863 015	46 115 233 530	251	Yes
Saccharum spontaneum	SRR486146	NC_034802	63 408 401	19 149 337 102	151	Yes
Salix purpurea	SRR3927005	NC_026722	191 948 456	57 584 536 800	150	Yes
Salix suchowensis	SRR8255647	NC_026462	53 495 983	16 048 794 900	150	Yes
Salvia miltiorrhiza	SRR1735356	NC_020431	215 771 972	43 585 938 344	101	NO
Sanionia uncinata	SRR6440975	NC_025668	3 961 386	2 379 651 487	300	NO

Continued on next page

Table S2 – *Continued from previous page*

Taxon	SRA Acc	Ref Acc	read pairs	base pairs (bp)	read len. (bp)	Consistency check
Schizachyrium scoparium	SRR6442327	NC_035032	1 476 472	442 941 600	150	Yes
Sciadopitys verticillata	ERR2799544	NC_029734	1 136 673	343 275 246	151	Yes
Scutellaria baicalensis	SRR6940088	NC_027262	27 930 422	8 379 126 600	150	Yes
Scutellaria lateriflora	SRR5602584	NC_034693	1 699 048	843 357 767	248	NO
Sequoia sempervirens	SRR1951920	NC_030372	2 354 579	1 412 747 400	300	NO
Sesamum indicum	SRR3407090	NC_016433	20 637 541	5 159 385 250	125	NO
Setaria italica	ERR2696316	NC_022850	65 844 392	19 753 317 600	150	Yes
Setaria viridis	SRR4063259	NC_028075	106 716 486	33 508 976 604	157	Yes
Silene latifolia	SRR7040334	NC_016730	284 434 421	71 108 605 250	125	NO
Solanum berthaultii	SRR5349620	NC_034951	30 261 020	7 565 255 000	125	NO
Solanum commersonii	SRR5349609	NC_028069	31 268 502	7 817 125 500	125	NO
Solanum habrochaites	DRR098098	NC_026879	95 529 750	17 768 533 500	93	NO
Solanum lycopersicum	DRR022700	AC_000188	4 192 871	2 104 821 242	251	Yes
Solanum melongena	DRR014074	NC_030207	173 060 640	34 958 249 280	101	NO
Solanum peruvianum	SRR1291239	NC_026881	12 772 339	2 449 431 901	96	Yes
Solanum pimpinellifolium	SRR074949	NC_026882	195 678 316	39 527 019 832	101	Yes
Solanum tuberosum	ERR3148763	NC_008096	806 926	243 691 652	151	NO
Sorghum bicolor	SRR3927127	NC_008602	30 065 772	9 079 863 144	151	NO
Sorghum timorense	SRR424217	NC_023800	200 900 821	40 180 164 200	100	Yes
Spirodela polyrhiza	SRR7548934	NC_015891	87 602 747	26 401 985 035	151	Yes
Sporobolus michauxianus	SRR4434178	NC_029416	183 439 649	55 031 894 700	150	NO
Stachys byzantina	SRR3170744	NC_029825	212 918	64 301 236	151	Yes
Stachys chamissonis	SRR3170745	NC_029822	2 570 411	776 264 122	151	Yes
Stachys coccinea	SRR3170746	NC_029823	292 594	88 363 388	151	Yes
Stachys sylvatica	SRR3170747	NC_029824	592 661	178 983 622	151	NO
Taraxacum kok-saghyz	SRR8185394	NC_032057	21 183 799	11 049 748 919	261	NO
Taxodium distichum	ERR2799546	NC_034941	879 574	265 631 348	151	Yes

Continued on next page

Table S2 – *Continued from previous page*

Taxon	SRA Acc	Ref Acc	read pairs	base pairs (bp)	read len. (bp)	Consistency check
Taxus baccata	ERR268425	NC_035066	172 310 721	34 462 144 200	100	Yes
Tectona grandis	SRR6940065	NC_020098	24 194 338	7 258 301 400	150	Yes
Tetradesmus obliquus	ERR1683652	NC_008101	100 944 122	20 390 712 644	101	NO
Themeda triandra	SRR7529014	NC_035016	91 131 777	45 565 888 500	250	Yes
Theobroma cacao	SRR5602583	NC_014676	470 765	277 504 475	295	NO
Thlaspi arvense	SRR1034659	NC_034362	10 761 690	5 402 368 380	251	NO
Trifolium subterraneum	DRR032042	NC_011828	25 964 880	15 630 857 760	301	Yes
Triticum aestivum	SRR5893651	NC_002762	197 767 716	104 816 889 480	265	NO
Triticum monococcum	SRR384895	NC_021760	157 945 836	31 905 058 872	101	NO
Triticum urartu	SRR4010671	NC_021762	123 772 698	63 619 166 772	257	NO
Urochloa ruziziensis	SRR6816710	NC_030068	154 533 231	46 669 035 762	151	NO
Utricularia reniformis	SRR5349713	NC_029719	2 186 073	1 157 291 061	265	NO
Vaccinium macrocarpon	SRR1276173	NC_019616	30 540 329	9 223 179 358	151	NO
Vicia sativa	ERR413103	NC_027155	10 232 390	2 046 478 000	100	NO
Vigna unguiculata	SRR7125688	NC_018051	30 233 396	9 070 018 800	150	Yes
Vitis aestivalis	SRR5891909	NC_029454	17 094 973	5 128 491 900	150	Yes
Vitis amurensis	SRR5891950	NC_031383	21 559 227	6 467 768 100	150	NO
Vitis rotundifolia	SRR5627788	NC_023790	49 224 878	14 767 463 400	150	NO
Vitis vinifera	SRR7160359	NC_007957	8 416 372	8 416 372 000	500	NO
Viviania marifolia	ERR2799536	NC_023259	1 050 522	317 257 644	151	NO
Wisteria floribunda	SRR1265941	NC_027677	9 382 854	1 895 336 508	101	Yes
Wollemia nobilis	SRR1927951	NC_027235	116 590	59 624 823	256	NO
Zea mays	SRR5826129	NC_001666	172 800 442	89 856 229 840	260	Yes

Table S3: Docker images used in our benchmark setup.

Tool	Image name and tag	SHA256 Checksum
chloroExtractor	chloroextracteam/benchmark_chloroextractor:v2.0.0	8f49e03424b37e699c5fea9391f79dbb9f2d0dc550ce86c4c700f39deec2dacd
Chloroplast assembly protocol	chloroextracteam/benchmark_chloroplast_assembly_protocol:v2.0.1	452544e3826748af5b988de0cf402bcea6ff5eb17afdcb71931959d2f6270ca6
Fast-Plast	chloroextracteam/benchmark_fastplast:v2.0.0	a3d06a610f8340ba49c3ff3b27342534e2a5348b17caa4fd3c3f3d327243a272
GetOrganelle	chloroextracteam/benchmark_getorganelle:v2.0.0	2ed3a464a82025a196ea56c649d0b0a3472cef76994bb065d56054d93298d956
IOGA	chloroextracteam/benchmark_ioga:v2.0.0	4698a21c343c60290bbf16811165a654ff01e8fddeb41cd75d0771b7f45968c0
NOVOPlasty	chloroextracteam/benchmark_novoplasty:v2.0.0	106387bad4e8e5c53fb9d4c3bdd60cdddf05a47b52a38697369eeabb947c547c
ORG.Asm	chloroextracteam/benchmark_org-asm:v2.0.0	ff83677c97b7c4e346191b9e04a5162eeea2a8dfd9e60ff84067d33747868f60

Table S4: Performance metrics input size 25K

	program	threads	Peak CPU usage	Peak memory	Peak disk usage (GB)
1	CAP	1	113.32	0.31	0.06
2	CAP	2	166.60	0.26	0.05
3	CAP	4	305.99	0.26	0.05
4	CAP	8	569.12	0.37	0.06
5	CE	1	111.49	3.41	0.07
6	CE	2	199.12	3.41	0.07
7	CE	4	343.22	3.41	0.07
8	CE	8	509.51	3.41	0.07
9	Fast-Plast	1	118.31	0.77	0.30
10	Fast-Plast	2	213.57	0.77	0.31
11	Fast-Plast	4	379.27	0.77	0.32
12	Fast-Plast	8	657.20	0.77	0.35
13	GetOrganelle	1	111.39	0.38	0.05
14	GetOrganelle	2	170.30	0.32	0.05
15	GetOrganelle	4	242.27	0.31	0.06
16	GetOrganelle	8	346.98	0.31	0.07
17	IOGA	1	118.12	18.61	6.25
18	IOGA	2	224.74	17.88	6.26
19	IOGA	4	394.98	16.62	5.60
20	IOGA	8	733.11	21.76	11.13
21	NOVOPlasty	1	78.38	0.04	0.00
22	NOVOPlasty	2	86.64	0.04	0.00
23	NOVOPlasty	4	86.50	0.04	0.00
24	NOVOPlasty	8	87.32	0.04	0.00
25	org.ASM	1	112.08	0.79	0.03
26	org.ASM	2	115.44	0.79	0.03
27	org.ASM	4	105.09	0.79	0.03
28	org.ASM	8	105.78	0.79	0.03

Table S5: Performance metrics input size 250K

	program	threads	Peak CPU usage	Peak memory	Peak disk usage (GB)
1	CAP	1	114.38	0.90	0.65
2	CAP	2	204.39	0.93	0.65
3	CAP	4	337.09	0.97	0.65
4	CAP	8	652.75	1.08	0.65
5	CE	1	117.57	3.96	0.63
6	CE	2	230.35	3.96	0.63
7	CE	4	414.25	3.96	0.63
8	CE	8	755.76	3.97	0.63
9	Fast-Plast	1	118.81	1.40	0.78
10	Fast-Plast	2	234.12	2.25	0.78
11	Fast-Plast	4	425.89	2.32	0.79
12	Fast-Plast	8	845.94	2.52	0.79
13	GetOrganelle	1	117.94	1.07	0.40
14	GetOrganelle	2	227.05	1.13	0.40
15	GetOrganelle	4	403.35	1.08	0.40
16	GetOrganelle	8	747.20	1.03	0.39
17	IOGA	1	118.62	12.03	4.01
18	IOGA	2	234.09	9.71	4.01
19	IOGA	4	420.77	10.09	3.48
20	IOGA	8	842.57	19.21	9.45
21	NOVOPlasty	1	111.97	0.32	0.00
22	NOVOPlasty	2	115.07	0.32	0.00
23	NOVOPlasty	4	116.42	0.31	0.00
24	NOVOPlasty	8	115.86	0.31	0.00
25	org.ASM	1	118.10	0.93	0.06
26	org.ASM	2	161.76	0.93	0.06
27	org.ASM	4	163.01	0.93	0.06
28	org.ASM	8	160.74	0.93	0.06

Table S6: Performance metrics input size 2.5M

	program	threads	Peak CPU usage	Peak memory	Peak disk usage (GB)
1	CAP	1	118.80	6.83	6.59
2	CAP	2	209.22	7.58	6.59
3	CAP	4	357.36	7.25	6.59
4	CAP	8	683.71	6.99	6.59
5	CE	1	118.78	5.34	2.01
6	CE	2	234.82	5.34	2.01
7	CE	4	415.74	5.28	2.09
8	CE	8	844.95	5.34	2.01
9	Fast-Plast	1	119.24	6.80	5.80
10	Fast-Plast	2	237.34	6.98	5.82
11	Fast-Plast	4	443.27	8.02	5.69
12	Fast-Plast	8	872.07	8.87	5.69
13	GetOrganelle	1	118.91	5.25	3.31
14	GetOrganelle	2	235.18	5.26	3.31
15	GetOrganelle	4	420.10	5.44	3.31
16	GetOrganelle	8	845.13	6.07	3.31
17	IOGA	1	119.46	18.64	8.83
18	IOGA	2	237.22	21.24	10.19
19	IOGA	4	425.50	18.71	10.56
20	IOGA	8	857.29	25.23	16.51
21	NOVOPlasty	1	112.67	2.69	0.00
22	NOVOPlasty	2	117.77	2.58	0.00
23	NOVOPlasty	4	117.40	2.74	0.00
24	NOVOPlasty	8	117.28	2.74	0.00
25	org.ASM	1	118.73	2.63	0.37
26	org.ASM	2	184.53	2.63	0.37
27	org.ASM	4	177.03	2.63	0.37
28	org.ASM	8	176.25	2.63	0.37

Table S7: **Novel data set.**

SRA Accession	Depth	TaxID	Rank	Related References with Depth	Status
DRR053713	3	71243	order	NC_033350(3)	success
DRR057122	3	3650	family	NC_007144(3) NC_023544(3) NC_029484(3)<br>NC_031834(3) NC_032008(3) NC_033899(3)<br>NC_015983(4)	success
DRR106816	5	2231393	no rank	NC_009259(5) NC_013843(5) NC_016708(5)<br>NC_018051(5) NC_021091(5) NC_023090(5)<br>NC_025909(5) NC_027677(5) NC_029406(5)<br>NC_031429(5) NC_034742(5) NC_034743(5)<br>NC_034774(5) NC_002694(6) NC_003119(6)<br>NC_007942(6) NC_011163(6) NC_011828(6)<br>NC_014057(6) NC_014063(6) NC_021636(6)<br>NC_021645(6) NC_021646(6) NC_021647(6)<br>NC_021648(6) NC_021649(6) NC_021650(6)<br>NC_022868(6) NC_024034(6) NC_024035(6)<br>NC_024036(6) NC_024038(6) NC_024166(6)<br>NC_025743(6) NC_025744(6) NC_025745(6)<br>NC_027073(6) NC_027074(6) NC_027075(6)<br>NC_027076(6) NC_027077(6) NC_027078(6)<br>NC_027079(6) NC_027080(6) NC_027148(6)<br>NC_027149(6) NC_027150(6) NC_027151(6)<br>NC_027152(6) NC_027153(6) NC_027154(6)<br>NC_027155(6) NC_029828(6) NC_030329(6)<br>NC_032066(6) NC_032691(6) NC_034229(6)<br>NC_035228(6) NC_035229(6) NC_028171(7)	success
ERR1917165	5	4118	family	NC_009808(3) NC_026703(3) NC_031159(3)<br>NC_034670(3)	success

Continued on next page

Table S7 – *Continued from previous page*

SRA Accession	Depth	TaxID	Rank	Related References with Depth	Status
ERR2003066	3	721789	tribe	NC_018767(3) NC_019601(3) NC_019602(3) NC_034347(3) NC_015206(4) NC_018766(4)	success
ERR2744401	5	156152	family	NC_028519(3) NC_028520(3) NC_031345(3) NC_034688(3) NC_031153(4) NC_031344(4)	success
ERR2744402	4	156152	family	NC_028519(3) NC_028520(3) NC_031345(3) NC_034688(3) NC_031153(4) NC_031344(4)	success
ERR3089127	4	102810	tribe	NC_020092(3) NC_020320(3) NC_020607(3) NC_025910(3) NC_030785(3) NC_031399(3) NC_031400(3) NC_034683(3) NC_034851(3)	success

Continued on next page

Table S7 – *Continued from previous page*

SRA Accession	Depth	TaxID	Rank	Related References with Depth	Status
SRR2154064	4	4210	family	NC_027835(4) NC_034648(4) NC_007578(5)	success
				NC_007977(5) NC_010601(5) NC_013553(5)	
				NC_015543(5) NC_015621(5) NC_020092(5)	
				NC_020320(5) NC_020607(5) NC_023107(5)	
				NC_023108(5) NC_023109(5) NC_023110(5)	
				NC_023111(5) NC_023112(5) NC_023113(5)	
				NC_023114(5) NC_023833(5) NC_024286(5)	
				NC_025910(5) NC_027113(5) NC_027434(5)	
				NC_028005(5) NC_028006(5) NC_028027(5)	
				NC_029465(5) NC_030173(5) NC_030275(5)	
				NC_030772(5) NC_030773(5) NC_030783(5)	
				NC_030785(5) NC_031395(5) NC_031396(5)	
				NC_031399(5) NC_031400(5) NC_031815(5)	
				NC_031816(5) NC_031833(5) NC_031853(5)	
				NC_031898(5) NC_032056(5) NC_032057(5)	
				NC_034320(5) NC_034321(5) NC_034322(5)	
				NC_034323(5) NC_034324(5) NC_034325(5)	
				NC_034326(5) NC_034327(5) NC_034328(5)	
				NC_034683(5) NC_034810(5) NC_034811(5)	
				NC_034812(5) NC_034813(5) NC_034814(5)	
				NC_034815(5) NC_034816(5) NC_034817(5)	
				NC_034818(5) NC_034819(5) NC_034820(5)	
				NC_034821(5) NC_034822(5) NC_034823(5)	
				NC_034824(5) NC_034825(5) NC_034827(5)	
				NC_034828(5) NC_034829(5) NC_034830(5)	
				NC_034831(5) NC_034832(5) NC_034847(5)	
				NC_034848(5) NC_034849(5) NC_034850(5)	
				NC_034851(5) NC_034852(5) NC_034853(5)	
				NC_034854(5) NC_034856(5) NC_034857(5)	
				NC_034858(5) NC_034859(5) NC_034860(5)	
				NC_034861(5) NC_034862(5) NC_034864(5)	
				NC_034865(5) NC_034866(5) NC_034867(5)	
				NC_034868(5) NC_034869(5) NC_034870(5)	
				NC_034871(5) NC_034872(5) NC_034873(5)	
				NC_034874(5) NC_034875(5) NC_034876(5)	
				NC_034877(5) NC_034878(5) NC_034879(5)	
				NC_034880(5) NC_034881(5) NC_034882(5)	
				NC_034883(5) NC_034884(5) NC_034885(5)	

Table S7 – *Continued from previous page*

SRA Accession	Depth	TaxID	Rank	Related References with Depth	Status
SRR2176133	3	40550	order	NC_026786(3) NC_027659(3)	success

Continued on next page

Table S7 – *Continued from previous page*

SRA Accession	Depth	TaxID	Rank	Related References with Depth			Status
SRR2401791	4	91888	no rank	NC_016433(4)	NC_021449(4)	NC_023463(4)	success
				NC_025652(4)	NC_025653(4)	NC_029719(4)	
				NC_030212(4)	NC_031435(4)	NC_031436(4)	
				NC_008407(5)	NC_009808(5)	NC_013707(5)	
				NC_020098(5)	NC_021111(5)	NC_022859(5)	
				NC_023102(5)	NC_023115(5)	NC_023131(5)	
				NC_023132(5)	NC_023464(5)	NC_023465(5)	
				NC_024845(5)	NC_025641(5)	NC_025642(5)	
				NC_025651(5)	NC_025787(5)	NC_026202(5)	
				NC_026291(5)	NC_026703(5)	NC_027262(5)	
				NC_027838(5)	NC_027955(5)	NC_028519(5)	
				NC_028520(5)	NC_028533(5)	NC_029700(5)	
				NC_031159(5)	NC_031345(5)	NC_031437(5)	
				NC_031441(5)	NC_031442(5)	NC_031443(5)	
				NC_031444(5)	NC_031445(5)	NC_033534(5)	
				NC_034308(5)	NC_034309(5)	NC_034310(5)	
				NC_034311(5)	NC_034312(5)	NC_034670(5)	
				NC_034688(5)	NC_034691(5)	NC_034693(5)	
				NC_034694(5)	NC_034695(5)	NC_035000(5)	
				NC_001879(6)	NC_004561(6)	NC_007500(6)	
				NC_007602(6)	NC_007943(6)	NC_008096(6)	
				NC_015401(6)	NC_015604(6)	NC_015608(6)	
				NC_015623(6)	NC_016068(6)	NC_018117(6)	
				NC_018552(6)	NC_022451(6)	NC_024261(6)	
				NC_024292(6)	NC_025657(6)	NC_026551(6)	
				NC_026563(6)	NC_026567(6)	NC_026570(6)	
				NC_026574(6)	NC_026694(6)	NC_026726(6)	
				NC_026906(6)	NC_027099(6)	NC_027177(6)	
				NC_027441(6)	NC_027442(6)	NC_028007(6)	
				NC_028009(6)	NC_028069(6)	NC_028070(6)	
				NC_028614(6)	NC_029370(6)	NC_029746(6)	
				NC_029817(6)	NC_029818(6)	NC_029819(6)	
				NC_029820(6)	NC_029821(6)	NC_029822(6)	
				NC_029823(6)	NC_029824(6)	NC_029825(6)	
				NC_029833(6)	NC_030044(6)	NC_030056(6)	
				NC_030167(6)	NC_030168(6)	NC_030171(6)	
				NC_030177(6)	NC_030178(6)	NC_030185(6)	
				NC_030207(6)	NC_030282(6)	NC_030319(6)	

Table S7 – *Continued from previous page*

SRA Accession	Depth	TaxID	Rank	Related References with Depth	Status
SRR2531270	3	3650	family	NC_007144(3) NC_023544(3) NC_029484(3) NC_031834(3) NC_032008(3) NC_033899(3) NC_015983(4)	success
SRR2531276	4	3650	family	NC_007144(3) NC_023544(3) NC_029484(3) NC_031834(3) NC_032008(3) NC_033899(3) NC_015983(4)	success
SRR2531278	3	3650	family	NC_007144(3) NC_023544(3) NC_029484(3) NC_031834(3) NC_032008(3) NC_033899(3) NC_015983(4)	success
SRR2531280	3	3650	family	NC_007144(3) NC_023544(3) NC_029484(3) NC_031834(3) NC_032008(3) NC_033899(3) NC_015983(4)	success
SRR2531284	3	3650	family	NC_007144(3) NC_023544(3) NC_029484(3) NC_031834(3) NC_032008(3) NC_033899(3) NC_015983(4)	success
SRR2531289	3	3650	family	NC_007144(3) NC_023544(3) NC_029484(3) NC_031834(3) NC_032008(3) NC_033899(3) NC_015983(4)	success

Continued on next page

Table S7 – *Continued from previous page*

SRA Accession	Depth	TaxID	Rank	Related References with Depth	Status
SRR4124062	7	2231393	no rank	NC_009259(5) NC_013843(5) NC_016708(5)<br>NC_018051(5) NC_021091(5) NC_023090(5)<br>NC_025909(5) NC_027677(5) NC_029406(5)<br>NC_031429(5) NC_034742(5) NC_034743(5)<br>NC_034774(5) NC_002694(6) NC_003119(6)<br>NC_007942(6) NC_011163(6) NC_011828(6)<br>NC_014057(6) NC_014063(6) NC_021636(6)<br>NC_021645(6) NC_021646(6) NC_021647(6)<br>NC_021648(6) NC_021649(6) NC_021650(6)<br>NC_022868(6) NC_024034(6) NC_024035(6)<br>NC_024036(6) NC_024038(6) NC_024166(6)<br>NC_025743(6) NC_025744(6) NC_025745(6)<br>NC_027073(6) NC_027074(6) NC_027075(6)<br>NC_027076(6) NC_027077(6) NC_027078(6)<br>NC_027079(6) NC_027080(6) NC_027148(6)<br>NC_027149(6) NC_027150(6) NC_027151(6)<br>NC_027152(6) NC_027153(6) NC_027154(6)<br>NC_027155(6) NC_029828(6) NC_030329(6)<br>NC_032066(6) NC_032691(6) NC_034229(6)<br>NC_035228(6) NC_035229(6) NC_028171(7)	success

Continued on next page

Table S7 – *Continued from previous page*

SRA Accession	Depth	TaxID	Rank	Related References with Depth	Status
SRR4124063	6	2231393	no rank	NC_009259(5) NC_013843(5) NC_016708(5) NC_018051(5) NC_021091(5) NC_023090(5) NC_025909(5) NC_027677(5) NC_029406(5) NC_031429(5) NC_034742(5) NC_034743(5) NC_034774(5) NC_002694(6) NC_003119(6) NC_007942(6) NC_011163(6) NC_011828(6) NC_014057(6) NC_014063(6) NC_021636(6) NC_021645(6) NC_021646(6) NC_021647(6) NC_021648(6) NC_021649(6) NC_021650(6) NC_022868(6) NC_024034(6) NC_024035(6) NC_024036(6) NC_024038(6) NC_024166(6) NC_025743(6) NC_025744(6) NC_025745(6) NC_027073(6) NC_027074(6) NC_027075(6) NC_027076(6) NC_027077(6) NC_027078(6) NC_027079(6) NC_027080(6) NC_027148(6) NC_027149(6) NC_027150(6) NC_027151(6) NC_027152(6) NC_027153(6) NC_027154(6) NC_027155(6) NC_029828(6) NC_030329(6) NC_032066(6) NC_032691(6) NC_034229(6) NC_035228(6) NC_035229(6) NC_028171(7)	success

Continued on next page

Table S7 – *Continued from previous page*

SRA Accession	Depth	TaxID	Rank	Related References with Depth	Status
SRR4124078	6	2231393	no rank	NC_009259(5) NC_013843(5) NC_016708(5)<br>NC_018051(5) NC_021091(5) NC_023090(5)<br>NC_025909(5) NC_027677(5) NC_029406(5)<br>NC_031429(5) NC_034742(5) NC_034743(5)<br>NC_034774(5) NC_002694(6) NC_003119(6)<br>NC_007942(6) NC_011163(6) NC_011828(6)<br>NC_014057(6) NC_014063(6) NC_021636(6)<br>NC_021645(6) NC_021646(6) NC_021647(6)<br>NC_021648(6) NC_021649(6) NC_021650(6)<br>NC_022868(6) NC_024034(6) NC_024035(6)<br>NC_024036(6) NC_024038(6) NC_024166(6)<br>NC_025743(6) NC_025744(6) NC_025745(6)<br>NC_027073(6) NC_027074(6) NC_027075(6)<br>NC_027076(6) NC_027077(6) NC_027078(6)<br>NC_027079(6) NC_027080(6) NC_027148(6)<br>NC_027149(6) NC_027150(6) NC_027151(6)<br>NC_027152(6) NC_027153(6) NC_027154(6)<br>NC_027155(6) NC_029828(6) NC_030329(6)<br>NC_032066(6) NC_032691(6) NC_034229(6)<br>NC_035228(6) NC_035229(6) NC_028171(7)	success

Continued on next page

Table S7 – *Continued from previous page*

SRA Accession	Depth	TaxID	Rank	Related References with Depth	Status
SRR4124079	7	2231393	no rank	NC_009259(5) NC_013843(5) NC_016708(5)<br>NC_018051(5) NC_021091(5) NC_023090(5)<br>NC_025909(5) NC_027677(5) NC_029406(5)<br>NC_031429(5) NC_034742(5) NC_034743(5)<br>NC_034774(5) NC_002694(6) NC_003119(6)<br>NC_007942(6) NC_011163(6) NC_011828(6)<br>NC_014057(6) NC_014063(6) NC_021636(6)<br>NC_021645(6) NC_021646(6) NC_021647(6)<br>NC_021648(6) NC_021649(6) NC_021650(6)<br>NC_022868(6) NC_024034(6) NC_024035(6)<br>NC_024036(6) NC_024038(6) NC_024166(6)<br>NC_025743(6) NC_025744(6) NC_025745(6)<br>NC_027073(6) NC_027074(6) NC_027075(6)<br>NC_027076(6) NC_027077(6) NC_027078(6)<br>NC_027079(6) NC_027080(6) NC_027148(6)<br>NC_027149(6) NC_027150(6) NC_027151(6)<br>NC_027152(6) NC_027153(6) NC_027154(6)<br>NC_027155(6) NC_029828(6) NC_030329(6)<br>NC_032066(6) NC_032691(6) NC_034229(6)<br>NC_035228(6) NC_035229(6) NC_028171(7)	success

Continued on next page

Table S7 – *Continued from previous page*

SRA Accession	Depth	TaxID	Rank	Related References with Depth	Status
SRR4457826	4	102809	tribe	NC_027434(3) NC_034810(3) NC_034811(3)<br>NC_034812(3) NC_034813(3) NC_034814(3)<br>NC_034815(3) NC_034816(3) NC_034817(3)<br>NC_034818(3) NC_034819(3) NC_034820(3)<br>NC_034821(3) NC_034822(3) NC_034823(3)<br>NC_034824(3) NC_034825(3) NC_034827(3)<br>NC_034828(3) NC_034829(3) NC_034830(3)<br>NC_034831(3) NC_034832(3) NC_034847(3)<br>NC_034848(3) NC_034849(3) NC_034850(3)<br>NC_034852(3) NC_034853(3) NC_034854(3)<br>NC_034856(3) NC_034857(3) NC_034858(3)<br>NC_034859(3) NC_034860(3) NC_034861(3)<br>NC_034862(3) NC_034864(3) NC_034865(3)<br>NC_034866(3) NC_034867(3) NC_034868(3)<br>NC_034869(3) NC_034870(3) NC_034871(3)<br>NC_034872(3) NC_034873(3) NC_034874(3)<br>NC_034875(3) NC_034876(3) NC_034877(3)<br>NC_034878(3) NC_034879(3) NC_034880(3)<br>NC_034881(3) NC_034882(3) NC_034883(3)<br>NC_034884(3) NC_034885(3) NC_034886(3)<br>NC_034887(3) NC_034888(3) NC_034889(3)<br>NC_034891(3) NC_034892(3) NC_034893(3)<br>NC_034894(3) NC_034895(3) NC_034896(3)<br>NC_034897(3) NC_034898(3) NC_034899(3)<br>NC_034900(3) NC_034901(3) NC_034902(3)<br>NC_034903(3) NC_034996(3)	success

Continued on next page

Table S7 – *Continued from previous page*

SRA Accession	Depth	TaxID	Rank	Related References with Depth	Status
SRR4457835	4	102809	tribe	NC_027434(3) NC_034810(3) NC_034811(3) NC_034812(3) NC_034813(3) NC_034814(3) NC_034815(3) NC_034816(3) NC_034817(3) NC_034818(3) NC_034819(3) NC_034820(3) NC_034821(3) NC_034822(3) NC_034823(3) NC_034824(3) NC_034825(3) NC_034827(3) NC_034828(3) NC_034829(3) NC_034830(3) NC_034831(3) NC_034832(3) NC_034847(3) NC_034848(3) NC_034849(3) NC_034850(3) NC_034852(3) NC_034853(3) NC_034854(3) NC_034856(3) NC_034857(3) NC_034858(3) NC_034859(3) NC_034860(3) NC_034861(3) NC_034862(3) NC_034864(3) NC_034865(3) NC_034866(3) NC_034867(3) NC_034868(3) NC_034869(3) NC_034870(3) NC_034871(3) NC_034872(3) NC_034873(3) NC_034874(3) NC_034875(3) NC_034876(3) NC_034877(3) NC_034878(3) NC_034879(3) NC_034880(3) NC_034881(3) NC_034882(3) NC_034883(3) NC_034884(3) NC_034885(3) NC_034886(3) NC_034887(3) NC_034888(3) NC_034889(3) NC_034891(3) NC_034892(3) NC_034893(3) NC_034894(3) NC_034895(3) NC_034896(3) NC_034897(3) NC_034898(3) NC_034899(3) NC_034900(3) NC_034901(3) NC_034902(3) NC_034903(3) NC_034996(3)	success

Continued on next page

Table S7 – *Continued from previous page*

SRA Accession	Depth	TaxID	Rank	Related References with Depth	Status
SRR5195283	3	4055	order	NC_024292(5) NC_025657(5) NC_027441(5) NC_027442(5) NC_028009(5) NC_028614(5) NC_030319(5) NC_031155(5) NC_033354(5) NC_034698(5) NC_021423(6) NC_022431(6) NC_022432(6) NC_025655(6) NC_025656(6) NC_025658(6) NC_029459(6) NC_029460(6) NC_008535(7) NC_030053(7)	success
SRR5409224	5	2231393	no rank	NC_009259(5) NC_013843(5) NC_016708(5) NC_018051(5) NC_021091(5) NC_023090(5) NC_025909(5) NC_027677(5) NC_029406(5) NC_031429(5) NC_034742(5) NC_034743(5) NC_034774(5) NC_002694(6) NC_003119(6) NC_007942(6) NC_011163(6) NC_011828(6) NC_014057(6) NC_014063(6) NC_021636(6) NC_021645(6) NC_021646(6) NC_021647(6) NC_021648(6) NC_021649(6) NC_021650(6) NC_022868(6) NC_024034(6) NC_024035(6) NC_024036(6) NC_024038(6) NC_024166(6) NC_025743(6) NC_025744(6) NC_025745(6) NC_027073(6) NC_027074(6) NC_027075(6) NC_027076(6) NC_027077(6) NC_027078(6) NC_027079(6) NC_027080(6) NC_027148(6) NC_027149(6) NC_027150(6) NC_027151(6) NC_027152(6) NC_027153(6) NC_027154(6) NC_027155(6) NC_029828(6) NC_030329(6) NC_032066(6) NC_032691(6) NC_034229(6) NC_035228(6) NC_035229(6) NC_028171(7)	success

Continued on next page

Table S7 – *Continued from previous page*

SRA Accession	Depth	TaxID	Rank	Related References with Depth	Status
SRR5409225	4	2231393	no rank	NC_009259(5) NC_013843(5) NC_016708(5) NC_018051(5) NC_021091(5) NC_023090(5) NC_025909(5) NC_027677(5) NC_029406(5) NC_031429(5) NC_034742(5) NC_034743(5) NC_034774(5) NC_002694(6) NC_003119(6) NC_007942(6) NC_011163(6) NC_011828(6) NC_014057(6) NC_014063(6) NC_021636(6) NC_021645(6) NC_021646(6) NC_021647(6) NC_021648(6) NC_021649(6) NC_021650(6) NC_022868(6) NC_024034(6) NC_024035(6) NC_024036(6) NC_024038(6) NC_024166(6) NC_025743(6) NC_025744(6) NC_025745(6) NC_027073(6) NC_027074(6) NC_027075(6) NC_027076(6) NC_027077(6) NC_027078(6) NC_027079(6) NC_027080(6) NC_027148(6) NC_027149(6) NC_027150(6) NC_027151(6) NC_027152(6) NC_027153(6) NC_027154(6) NC_027155(6) NC_029828(6) NC_030329(6) NC_032066(6) NC_032691(6) NC_034229(6) NC_035228(6) NC_035229(6) NC_028171(7)	success
SRR5581778	4	3650	family	NC_007144(3) NC_023544(3) NC_029484(3) NC_031834(3) NC_032008(3) NC_033899(3) NC_015983(4)	success
SRR5586278	5	1521262	subclass	NC_012818(4) NC_022136(4) NC_022137(4) NC_024157(4) NC_024158(4) NC_004766(6) NC_014348(6) NC_014592(6) NC_028542(6) NC_028543(6) NC_028705(6)	success

Continued on next page

Table S7 – *Continued from previous page*

SRA Accession	Depth	TaxID	Rank	Related References with Depth	Status
SRR5880481	3	41945	order	NC_020019(3) NC_021121(3) NC_022264(3) NC_022459(3) NC_022460(3) NC_022461(3) NC_022462(3) NC_022463(3) NC_023084(3) NC_024541(3) NC_024543(3) NC_024659(3) NC_024660(3) NC_024661(3) NC_024662(3) NC_024663(3) NC_026197(3) NC_026690(3) NC_026691(3) NC_030180(3) NC_030539(3) NC_030609(3) NC_030784(3) NC_030786(3) NC_030787(3) NC_030789(3) NC_031186(3) NC_031187(3) NC_031428(3) NC_031892(3) NC_033502(3) NC_034331(3) NC_034371(3) NC_034640(3) NC_034641(3) NC_034677(3) NC_034678(3) NC_034913(3) NC_034914(3) NC_034915(3) NC_035418(3) NC_033501(4) NC_019616(5) NC_029704(5)	success
SRR6331521	3	4149	family	NC_026202(3) NC_031437(3)	success

Continued on next page

Table S7 – *Continued from previous page*

SRA Accession	Depth	TaxID	Rank	Related References with Depth	Status
SRR6425047	5	2231393	no rank	NC_009259(5) NC_013843(5) NC_016708(5)<br>NC_018051(5) NC_021091(5) NC_023090(5)<br>NC_025909(5) NC_027677(5) NC_029406(5)<br>NC_031429(5) NC_034742(5) NC_034743(5)<br>NC_034774(5) NC_002694(6) NC_003119(6)<br>NC_007942(6) NC_011163(6) NC_011828(6)<br>NC_014057(6) NC_014063(6) NC_021636(6)<br>NC_021645(6) NC_021646(6) NC_021647(6)<br>NC_021648(6) NC_021649(6) NC_021650(6)<br>NC_022868(6) NC_024034(6) NC_024035(6)<br>NC_024036(6) NC_024038(6) NC_024166(6)<br>NC_025743(6) NC_025744(6) NC_025745(6)<br>NC_027073(6) NC_027074(6) NC_027075(6)<br>NC_027076(6) NC_027077(6) NC_027078(6)<br>NC_027079(6) NC_027080(6) NC_027148(6)<br>NC_027149(6) NC_027150(6) NC_027151(6)<br>NC_027152(6) NC_027153(6) NC_027154(6)<br>NC_027155(6) NC_029828(6) NC_030329(6)<br>NC_032066(6) NC_032691(6) NC_034229(6)<br>NC_035228(6) NC_035229(6) NC_028171(7)	success

Continued on next page

Table S7 – *Continued from previous page*

SRA Accession	Depth	TaxID	Rank	Related References with Depth	Status
SRR6425048	5	2231393	no rank	NC_009259(5) NC_013843(5) NC_016708(5)<br>NC_018051(5) NC_021091(5) NC_023090(5)<br>NC_025909(5) NC_027677(5) NC_029406(5)<br>NC_031429(5) NC_034742(5) NC_034743(5)<br>NC_034774(5) NC_002694(6) NC_003119(6)<br>NC_007942(6) NC_011163(6) NC_011828(6)<br>NC_014057(6) NC_014063(6) NC_021636(6)<br>NC_021645(6) NC_021646(6) NC_021647(6)<br>NC_021648(6) NC_021649(6) NC_021650(6)<br>NC_022868(6) NC_024034(6) NC_024035(6)<br>NC_024036(6) NC_024038(6) NC_024166(6)<br>NC_025743(6) NC_025744(6) NC_025745(6)<br>NC_027073(6) NC_027074(6) NC_027075(6)<br>NC_027076(6) NC_027077(6) NC_027078(6)<br>NC_027079(6) NC_027080(6) NC_027148(6)<br>NC_027149(6) NC_027150(6) NC_027151(6)<br>NC_027152(6) NC_027153(6) NC_027154(6)<br>NC_027155(6) NC_029828(6) NC_030329(6)<br>NC_032066(6) NC_032691(6) NC_034229(6)<br>NC_035228(6) NC_035229(6) NC_028171(7)	success

Continued on next page

Table S7 – *Continued from previous page*

SRA Accession	Depth	TaxID	Rank	Related References with Depth	Status
SRR6425049	5	2231393	no rank	NC_009259(5) NC_013843(5) NC_016708(5) NC_018051(5) NC_021091(5) NC_023090(5) NC_025909(5) NC_027677(5) NC_029406(5) NC_031429(5) NC_034742(5) NC_034743(5) NC_034774(5) NC_002694(6) NC_003119(6) NC_007942(6) NC_011163(6) NC_011828(6) NC_014057(6) NC_014063(6) NC_021636(6) NC_021645(6) NC_021646(6) NC_021647(6) NC_021648(6) NC_021649(6) NC_021650(6) NC_022868(6) NC_024034(6) NC_024035(6) NC_024036(6) NC_024038(6) NC_024166(6) NC_025743(6) NC_025744(6) NC_025745(6) NC_027073(6) NC_027074(6) NC_027075(6) NC_027076(6) NC_027077(6) NC_027078(6) NC_027079(6) NC_027080(6) NC_027148(6) NC_027149(6) NC_027150(6) NC_027151(6) NC_027152(6) NC_027153(6) NC_027154(6) NC_027155(6) NC_029828(6) NC_030329(6) NC_032066(6) NC_032691(6) NC_034229(6) NC_035228(6) NC_035229(6) NC_028171(7)	success
SRR6425628	4	167487	subfamily	NC_024292(3) NC_033354(3) NC_021423(4)	success
SRR6425646	4	167487	subfamily	NC_024292(3) NC_033354(3) NC_021423(4)	success
SRR6425647	4	167487	subfamily	NC_024292(3) NC_033354(3) NC_021423(4)	success
SRR6425648	4	167487	subfamily	NC_024292(3) NC_033354(3) NC_021423(4)	success
SRR6425651	4	167483	subfamily	NC_025657(3) NC_025655(4) NC_025656(4) NC_025658(4)	success

Continued on next page

Table S7 – *Continued from previous page*

SRA Accession	Depth	TaxID	Rank	Related References with Depth	Status
SRR6513658	3	3814	subfamily	NC_009259(6) NC_013843(6) NC_016708(6)<br>NC_018051(6) NC_021091(6) NC_023090(6)<br>NC_025909(6) NC_027677(6) NC_029406(6)<br>NC_031429(6) NC_034742(6) NC_034743(6)<br>NC_034774(6) NC_002694(7) NC_003119(7)<br>NC_007942(7) NC_011163(7) NC_011828(7)<br>NC_014057(7) NC_014063(7) NC_021636(7)<br>NC_021645(7) NC_021646(7) NC_021647(7)<br>NC_021648(7) NC_021649(7) NC_021650(7)<br>NC_022868(7) NC_024034(7) NC_024035(7)<br>NC_024036(7) NC_024038(7) NC_024166(7)<br>NC_025743(7) NC_025744(7) NC_025745(7)<br>NC_027073(7) NC_027074(7) NC_027075(7)<br>NC_027076(7) NC_027077(7) NC_027078(7)<br>NC_027079(7) NC_027080(7) NC_027148(7)<br>NC_027149(7) NC_027150(7) NC_027151(7)<br>NC_027152(7) NC_027153(7) NC_027154(7)<br>NC_027155(7) NC_029828(7) NC_030329(7)<br>NC_032066(7) NC_032691(7) NC_034229(7)<br>NC_035228(7) NC_035229(7) NC_028171(8)	success

Continued on next page

Table S7 – *Continued from previous page*

SRA Accession	Depth	TaxID	Rank	Related References with Depth	Status
SRR6821984	3	3700	family	NC_000932(3) NC_009265(3) NC_009266(3) NC_009267(3) NC_009268(3) NC_009269(3) NC_009270(3) NC_009271(3) NC_009272(3) NC_009273(3) NC_009274(3) NC_009275(3) NC_016734(3) NC_018565(3) NC_021102(3) NC_023367(3) NC_024469(3) NC_026445(3) NC_026446(3) NC_027693(3) NC_028170(3) NC_028272(3) NC_028415(3) NC_028517(3) NC_028726(3) NC_028727(3) NC_028728(3) NC_029253(3) NC_029254(3) NC_029331(3) NC_029332(3) NC_029333(3) NC_029334(3) NC_029335(3) NC_029336(3) NC_029337(3) NC_029378(3) NC_029379(3) NC_030346(3) NC_030347(3) NC_030348(3) NC_030349(3) NC_030350(3) NC_030351(3) NC_030450(3) NC_030515(3) NC_030516(3) NC_030775(3) NC_033499(3) NC_033500(3) NC_034287(3) NC_034299(3) NC_034357(3) NC_034359(3) NC_034360(3) NC_034361(3) NC_034362(3) NC_034363(3) NC_034365(3) NC_034366(3) NC_034367(3) NC_035303(3) NC_034286(4) NC_034379(4)	success

Continued on next page

Table S7 – *Continued from previous page*

SRA Accession	Depth	TaxID	Rank	Related References with Depth	Status
SRR6940055	3	4136	family	NC_020098(3) NC_023102(3) NC_026291(3)<br>NC_027262(3) NC_028533(3) NC_034693(3)<br>NC_029370(4) NC_029817(4) NC_029818(4)<br>NC_029819(4) NC_029820(4) NC_029821(4)<br>NC_029822(4) NC_029823(4) NC_029824(4)<br>NC_029825(4) NC_030755(4) NC_030756(4)<br>NC_030757(4) NC_031433(4) NC_031434(4)<br>NC_031874(4) NC_032054(4) NC_035143(4)<br>NC_020431(5) NC_027259(5) NC_035233(5)	success
SRR6940081	3	4136	family	NC_020098(3) NC_023102(3) NC_026291(3)<br>NC_027262(3) NC_028533(3) NC_034693(3)<br>NC_029370(4) NC_029817(4) NC_029818(4)<br>NC_029819(4) NC_029820(4) NC_029821(4)<br>NC_029822(4) NC_029823(4) NC_029824(4)<br>NC_029825(4) NC_030755(4) NC_030756(4)<br>NC_030757(4) NC_031433(4) NC_031434(4)<br>NC_031874(4) NC_032054(4) NC_035143(4)<br>NC_020431(5) NC_027259(5) NC_035233(5)	success

Continued on next page

Table S7 – *Continued from previous page*

SRA Accession	Depth	TaxID	Rank	Related References with Depth	Status
SRR7223704	3	3700	family	NC_000932(3) NC_009265(3) NC_009266(3) NC_009267(3) NC_009268(3) NC_009269(3) NC_009270(3) NC_009271(3) NC_009272(3) NC_009273(3) NC_009274(3) NC_009275(3) NC_016734(3) NC_018565(3) NC_021102(3) NC_023367(3) NC_024469(3) NC_026445(3) NC_026446(3) NC_027693(3) NC_028170(3) NC_028272(3) NC_028415(3) NC_028517(3) NC_028726(3) NC_028727(3) NC_028728(3) NC_029253(3) NC_029254(3) NC_029331(3) NC_029332(3) NC_029333(3) NC_029334(3) NC_029335(3) NC_029336(3) NC_029337(3) NC_029378(3) NC_029379(3) NC_030346(3) NC_030347(3) NC_030348(3) NC_030349(3) NC_030350(3) NC_030351(3) NC_030450(3) NC_030515(3) NC_030516(3) NC_030775(3) NC_033499(3) NC_033500(3) NC_034287(3) NC_034299(3) NC_034357(3) NC_034359(3) NC_034360(3) NC_034361(3) NC_034362(3) NC_034363(3) NC_034365(3) NC_034366(3) NC_034367(3) NC_035303(3) NC_034286(4) NC_034379(4)	success

Continued on next page

Table S7 – *Continued from previous page*

SRA Accession	Depth	TaxID	Rank	Related References with Depth	Status
SRR7223710	3	3700	family	NC_000932(3) NC_009265(3) NC_009266(3) NC_009267(3) NC_009268(3) NC_009269(3) NC_009270(3) NC_009271(3) NC_009272(3) NC_009273(3) NC_009274(3) NC_009275(3) NC_016734(3) NC_018565(3) NC_021102(3) NC_023367(3) NC_024469(3) NC_026445(3) NC_026446(3) NC_027693(3) NC_028170(3) NC_028272(3) NC_028415(3) NC_028517(3) NC_028726(3) NC_028727(3) NC_028728(3) NC_029253(3) NC_029254(3) NC_029331(3) NC_029332(3) NC_029333(3) NC_029334(3) NC_029335(3) NC_029336(3) NC_029337(3) NC_029378(3) NC_029379(3) NC_030346(3) NC_030347(3) NC_030348(3) NC_030349(3) NC_030350(3) NC_030351(3) NC_030450(3) NC_030515(3) NC_030516(3) NC_030775(3) NC_033499(3) NC_033500(3) NC_034287(3) NC_034299(3) NC_034357(3) NC_034359(3) NC_034360(3) NC_034361(3) NC_034362(3) NC_034363(3) NC_034365(3) NC_034366(3) NC_034367(3) NC_035303(3) NC_034286(4) NC_034379(4)	success

Continued on next page

Table S7 – *Continued from previous page*

SRA Accession	Depth	TaxID	Rank	Related References with Depth	Status
SRR8082590	3	3646	order	NC_024060(3) NC_024061(3) NC_024062(3)<br>NC_024063(3) NC_024064(3) NC_024065(3)<br>NC_024066(3) NC_024067(3) NC_026986(3)<br>NC_030517(3) NC_030534(3) NC_030544(3)<br>NC_030545(3) NC_030546(3) NC_030547(3)<br>NC_030548(3) NC_030549(3) NC_030550(3)<br>NC_030551(3) NC_030552(3) NC_030553(3)<br>NC_030554(3) NC_030555(3) NC_030556(3)<br>NC_030557(3) NC_030558(3) NC_030559(3)<br>NC_030560(3) NC_030561(3) NC_030562(3)<br>NC_030563(3) NC_030564(3) NC_030565(3)<br>NC_030566(3) NC_030567(3) NC_030568(3)<br>NC_030569(3) NC_030570(3) NC_030571(3)<br>NC_030572(3) NC_030573(3) NC_030574(3)<br>NC_030575(3) NC_030576(3) NC_030577(3)<br>NC_030578(3) NC_030579(3) NC_030580(3)<br>NC_030581(3) NC_030582(3) NC_030583(3)<br>NC_030601(3) NC_034285(3) NC_008235(4)<br>NC_009143(4) NC_024681(4) NC_024734(4)<br>NC_024735(4) NC_024747(4) NC_026462(4)<br>NC_026722(4) NC_027425(4) NC_028350(4)<br>NC_028504(4) NC_031371(4) NC_031398(4)<br>NC_032060(4) NC_032368(4) NC_032717(4)<br>NC_033876(4) NC_010433(5) NC_012224(5)<br>NC_015308(5) NC_016736(5) NC_034803(5)<br>NC_033910(7)	success

Continued on next page

Table S7 – *Continued from previous page*

SRA Accession	Depth	TaxID	Rank	Related References with Depth	Status
SRR8481357	5	2231393	no rank	NC_009259(5) NC_013843(5) NC_016708(5) NC_018051(5) NC_021091(5) NC_023090(5) NC_025909(5) NC_027677(5) NC_029406(5) NC_031429(5) NC_034742(5) NC_034743(5) NC_034774(5) NC_002694(6) NC_003119(6) NC_007942(6) NC_011163(6) NC_011828(6) NC_014057(6) NC_014063(6) NC_021636(6) NC_021645(6) NC_021646(6) NC_021647(6) NC_021648(6) NC_021649(6) NC_021650(6) NC_022868(6) NC_024034(6) NC_024035(6) NC_024036(6) NC_024038(6) NC_024166(6) NC_025743(6) NC_025744(6) NC_025745(6) NC_027073(6) NC_027074(6) NC_027075(6) NC_027076(6) NC_027077(6) NC_027078(6) NC_027079(6) NC_027080(6) NC_027148(6) NC_027149(6) NC_027150(6) NC_027151(6) NC_027152(6) NC_027153(6) NC_027154(6) NC_027155(6) NC_029828(6) NC_030329(6) NC_032066(6) NC_032691(6) NC_034229(6) NC_035228(6) NC_035229(6) NC_028171(7)	success

Continued on next page

Table S7 – *Continued from previous page*

SRA Accession	Depth	TaxID	Rank	Related References with Depth	Status
SRR8481359	5	2231393	no rank	NC_009259(5) NC_013843(5) NC_016708(5)<br>NC_018051(5) NC_021091(5) NC_023090(5)<br>NC_025909(5) NC_027677(5) NC_029406(5)<br>NC_031429(5) NC_034742(5) NC_034743(5)<br>NC_034774(5) NC_002694(6) NC_003119(6)<br>NC_007942(6) NC_011163(6) NC_011828(6)<br>NC_014057(6) NC_014063(6) NC_021636(6)<br>NC_021645(6) NC_021646(6) NC_021647(6)<br>NC_021648(6) NC_021649(6) NC_021650(6)<br>NC_022868(6) NC_024034(6) NC_024035(6)<br>NC_024036(6) NC_024038(6) NC_024166(6)<br>NC_025743(6) NC_025744(6) NC_025745(6)<br>NC_027073(6) NC_027074(6) NC_027075(6)<br>NC_027076(6) NC_027077(6) NC_027078(6)<br>NC_027079(6) NC_027080(6) NC_027148(6)<br>NC_027149(6) NC_027150(6) NC_027151(6)<br>NC_027152(6) NC_027153(6) NC_027154(6)<br>NC_027155(6) NC_029828(6) NC_030329(6)<br>NC_032066(6) NC_032691(6) NC_034229(6)<br>NC_035228(6) NC_035229(6) NC_028171(7)	success

Continued on next page

Table S7 – *Continued from previous page*

SRA Accession	Depth	TaxID	Rank	Related References with Depth	Status
SRR8784049	7	2231393	no rank	NC_009259(5) NC_013843(5) NC_016708(5) NC_018051(5) NC_021091(5) NC_023090(5) NC_025909(5) NC_027677(5) NC_029406(5) NC_031429(5) NC_034742(5) NC_034743(5) NC_034774(5) NC_002694(6) NC_003119(6) NC_007942(6) NC_011163(6) NC_011828(6) NC_014057(6) NC_014063(6) NC_021636(6) NC_021645(6) NC_021646(6) NC_021647(6) NC_021648(6) NC_021649(6) NC_021650(6) NC_022868(6) NC_024034(6) NC_024035(6) NC_024036(6) NC_024038(6) NC_024166(6) NC_025743(6) NC_025744(6) NC_025745(6) NC_027073(6) NC_027074(6) NC_027075(6) NC_027076(6) NC_027077(6) NC_027078(6) NC_027079(6) NC_027080(6) NC_027148(6) NC_027149(6) NC_027150(6) NC_027151(6) NC_027152(6) NC_027153(6) NC_027154(6) NC_027155(6) NC_029828(6) NC_030329(6) NC_032066(6) NC_032691(6) NC_034229(6) NC_035228(6) NC_035229(6) NC_028171(7)	success

Continued on next page

Table S7 – *Continued from previous page*

SRA Accession	Depth	TaxID	Rank	Related References with Depth	Status
SRR8784093	5	2231393	no rank	NC_009259(5) NC_013843(5) NC_016708(5)<br>NC_018051(5) NC_021091(5) NC_023090(5)<br>NC_025909(5) NC_027677(5) NC_029406(5)<br>NC_031429(5) NC_034742(5) NC_034743(5)<br>NC_034774(5) NC_002694(6) NC_003119(6)<br>NC_007942(6) NC_011163(6) NC_011828(6)<br>NC_014057(6) NC_014063(6) NC_021636(6)<br>NC_021645(6) NC_021646(6) NC_021647(6)<br>NC_021648(6) NC_021649(6) NC_021650(6)<br>NC_022868(6) NC_024034(6) NC_024035(6)<br>NC_024036(6) NC_024038(6) NC_024166(6)<br>NC_025743(6) NC_025744(6) NC_025745(6)<br>NC_027073(6) NC_027074(6) NC_027075(6)<br>NC_027076(6) NC_027077(6) NC_027078(6)<br>NC_027079(6) NC_027080(6) NC_027148(6)<br>NC_027149(6) NC_027150(6) NC_027151(6)<br>NC_027152(6) NC_027153(6) NC_027154(6)<br>NC_027155(6) NC_029828(6) NC_030329(6)<br>NC_032066(6) NC_032691(6) NC_034229(6)<br>NC_035228(6) NC_035229(6) NC_028171(7)	success
