# Supplementary material for: A systematic comparison of chloroplast genome assembly tools
Source: Genome Biol. 2020 Sep 28;21:254. doi: 10.1186/s13059-020-02153-6 (PMC7520963; doi:10.1186/s13059-020-02153-6)
Supplement: Supplementary file 2 — Additional file 2 Review history. [file 13059_2020_2153_MOESM2_ESM.docx]

Review History

**First round of review**

**Reviewer 1**

**Are you able to assess all statistics in the manuscript, including the appropriateness of statistical tests used?**

No, I do not feel adequately qualified to assess the statistics.

**Comments to author:**

Methods are appropriate but same authors developed ChloroExtractor, an important tool used as a benchmark in this study.  Authors compare seven chloroplast genome assembly tools.  So, this reviewer is not sure of novelty of this manuscript.  So, authors haven't addressed the important question "Does the work represent a significant advance over previously published studies?".  
User experience and success rate should be determined by users and documented through satisfaction surveys.  This is especially important when authors emphasize that program can be used using standard computer infrastructure.
There is >1,000 sequenced chloroplast genomes, with a large majority not fully assembled or annotated.  So, authors have plenty of opportunity to demonstrate use of these new assembly tools.
It is well known that intergenic spacer regions are not conserved and not even a single intergenic spacer region is conserved within chloroplasts genomes of the graminae or grass genomes.  Intergenic spacer region occupies >50% of the genome.  So, how does reference genome help in genome assembly?

In order to have real life comparison, authors could have sequenced few chloroplast genomes and used that data to compare different assembly tools and time taken for each program, pros and cons.
- Is the paper of broad interest to others in the field, or of outstanding interest to a broad audience of biologists?
This is of interest to evolutionary biologists to study phylogenetics or for chloroplast genetic engineering.

**Reviewer 2**

**Are you able to assess all statistics in the manuscript, including the appropriateness of statistical tests used?**

No, I do not feel adequately qualified to assess the statistics

**Comments to author:**

This study provides an systematically comparison of the chloroplast assembly tools at first and show significant differences between the tested assemblers in terms of generating whole chloroplast genome sequences and computational requirements. Moreover, the authors created docker images for each tested tool, which are available for the scientific community and improved reproducibility, thus large scale screening for chloroplasts as hidden treasures within genomic sequencing data is feasible.
   This topic of the review paper is knowledgeable and helpful for all the scientists who are working in the field of plant chloroplast assembly. However, the overall manuscript is not well written and contains limited information about systematically comparison of the chloroplast assembly tools and docker images for assembled reproducibility.

Specific points:

The number of lines on the edge does not correspond to the maintext. The major concerns about this manuscript can be found below and major revision is suggested before publication in "Genome Biology".
Page1  part of "General introduction and motivation"
Line 4: It should be quote recent article
Line 11: It should be quote recent article
Page2
Line 16: It should be IRa and IRb
Line 18: It should be quote recent article
Line 24-27: Re-write sentence
Part of "Approaches to extracting chloroplasts from whole genome data"
Line 6: Delete the dot before the quote
Lines 7-9: Re-write sentence
Part of "Purpose and scope of this study"
Line 2: It should be in italic type
Line 2: It should be genome
Line 3: Raw genomic data sets should be filter out low-quality reads by quality control
Lines 4 and 5: Many command-line tools of de novo assemble for chloroplast genome have all of these features, e.g., MITObim.
Part of "Time requirements"
Line 1: It should be among
Line 4: Different tools with more than 1 gigabyte data sets should be tested, and Figure1 can`t give valuable information as no significant difference for time-consuming. The same question for Part of "Memory and CPU Usage". Why these seven assemblers were chosen to test? Figure should be "Fig." and table as "Table".
Part of "Qualitative"
Line 3: NGS data for crop genomes can`t be used for GetOrganelle? In this part, the same data set should be used to test for every assemblers better.
Part of "Quantitative"  
Part of "Simulated data"
Line 1: Why not show the result for different data sets with every assemblers in Fig.3 and Table 2? Fig.3 and table 2 show no regular pattern in scores except the assembler GetOrganelle obtaining perfect.
Part of "Real data sets"
Line 3: The performance of GetOrganelle was bad, as only 199 circular assemblies out of a total of 356 assemblies that resulted in an output, the percent is 55.9%, however it achieved a median scores of 99.7. The number "99.7" should to be "89.13", as show in table 3? The SD value of GetOrganelle is too large than the other three tools but it achieved the highest scores.
Part of "Consistency"
Line 3: As shown in the Fig.6, only two tools achieved R2  not equal 1 by re-running assemblies and comparison of the scores of two assemblers, however, GetOrganelle defined as the only tool that succeed in obtaining similar score for all assemblers, this conclusion is not rigorous

**Authors Response**

**Point-by-point responses to the reviewers’ comments:**

**Reviewer 1**

Methods are appropriate but same authors developed ChloroExtractor, an important tool used as a benchmark in this study. Authors compare seven chloroplast genome assembly tools. So, this reviewer is not sure of novelty of this manuscript. So, authors haven’t addressed the important question "Does the work represent a significant advance over previously published studies?".

*Indeed the novelty in this article is not a new approach to the task of chloroplast genome assembly but rather a thorough benchmark of existing tools (including our tool ChloroExtractor). This represents a substantial advance for the field as there are competing tools published for the same task without any information about their relative performances. Currently, researchers have to do this evaluation of tools themselves to find the appropriate tool which leads to duplicated efforts as this is done by every group separately and without sharing results. If this evaluation is neglected a non optimal tool might be used which can lead to additional manual efforts in finishing or additional costs if more data is generated. Thus, clear guidelines to the end user (and important directions for improvement to the developers) are novel, important and beneficial for the research community.*

User experience and success rate should be determined by users and documented through satisfaction surveys. This is especially important when authors emphasize that program can be used using standard computer infrastructure.

*We agree that this would be the best way to measure and quantify user experience. However, the main focus of the work was on quantitative performance measures. Nevertheless, we decided to include usability metrics that were obtainable in a defined and structured way using the reviewer guidelines of the “Journal of Open Source Software” (JOSS).*

There is >1,000 sequenced chloroplast genomes, with a large majority not fully assembled or annotated. So, authors have plenty of opportunity to demonstrate use of these new assembly tools.

*Thanks for pointing this out. We now include as a proof of principle the application of these tools on 105 novel data sets. The assembly of 49 of those 105 data sets returned chloroplast genomes. After further screening against NCBI databases, still 20 data sets remained. That sets contained new chloroplast genomes for species without a reported plastid genome. We thus clearly demonstrate that the insights from this article can be used to produce further novel results that can lead to further biological insight.*

It is well known that intergenic spacer regions are not conserved and not even a single intergenic spacer region is conserved within chloroplasts genomes of the graminae or grass genomes. Intergenic spacer region occupies >50% of the genome. So, how does reference genome help in genome assembly?

*This is a very interesting question. But due to we are comparing de novo assembly tools, we think answering this question is outside the scope of our publication. Nevertheless, using reference plastid genomes, is helpful to sort the huge amount of input data into potential plastid genome related sequences and non related sequences which need no longer to be considered for an assembly.*

In order to have real life comparison, authors could have sequenced few chloroplast genomes and used that data to compare different assembly tools and time taken for each program, pros and cons.

*We already tested the tools on real data sets downloaded from NCBIs SRA which is equivalent to customly sequenced data. But we now include additional 105 novel data sets with no reference chloroplast in the database.*

- Is the paper of broad interest to others in the field, or of outstanding interest to a broad audience of biologists? This is of interest to evolutionary biologists to study phylogenetics or for chloroplast genetic engineering.

**Reviewer 2**

This study provides an systematically comparison of the chloroplast assembly tools at first and show significant differences between the tested assemblers in terms of generating whole chloroplast genome sequences and computational requirements. Moreover, the authors created docker images for each tested tool, which are available for the scientific community and improved reproducibility, thus large scale screening for chloroplasts as hidden treasures within genomic sequencing data is feasible. This topic of the review paper is knowledgeable and helpful for all the scientists who are working in the field of plant chloroplast assembly. However, the overall manuscript is not well written and contains limited information about systematically comparison of the chloroplast assembly tools and docker images for assembled reproducibility.

*Thanks for your thorough review and the pointers that helped us improve the overall readability of the manuscript. In addition we received feedback and edits from a native English speaker to further improve readability. We try to add more information about our systematically comparison into the manuscript, moreover, we provide all our script and even the docker container in our GitHub repository, DockerHub, and Zenodo to enable a maximum of reproducibility.*

Specific points: The number of lines on the edge does not correspond to the maintext. The major concerns about this manuscript can be found below and major revision is suggested before publication in "Genome Biology".

Page1 part of "General introduction and motivation"

Line 4: It should be quote recent article

Line 11: It should be quote recent article

Page2

Line 16: It should be IRa and IRb

Line 18: It should be quote recent article

Line 24-27: Re-write sentence

Part of "Approaches to extracting chloroplasts from whole genome data"

Line 6: Delete the dot before the quote

Lines 7-9: Re-write sentence

Part of "Purpose and scope of this study"

Line 2: It should be in italic type

Line 2: It should be genome

Line 3: Raw genomic data sets should be filter out low-quality reads by quality control

Lines 4 and 5: Many command-line tools of de novo assemble for chloroplast genome have all of these features, e.g., MITObim.

Part of "Time requirements"

Line 1: It should be among

Line 4: Different tools with more than 1 gigabyte data sets should be tested, and Figure1 can‘t give valuable information as no significant difference for time-consuming. The same question for Part of "Memory and CPU Usage". Why these seven assemblers were chosen to test? Figure should be "Fig." and table as "Table".

Part of "Qualitative"

Line 3: NGS data for crop genomes can‘t be used for GetOrganelle? In this part, the same data set should be used to test for every assemblers better.

Part of "Quantitative"

Part of "Simulated data"

Line 1: Why not show the result for different data sets with every assemblers in Fig.3 and Table 2? Fig.3 and table 2 show no regular pattern in scores except the assembler GetOrganelle obtaining perfect.

Part of "Real data sets"

Line 3: The performance of GetOrganelle was bad, as only 199 circular assemblies out of a total of 356 assemblies that resulted in an output, the percent is 55.9%, however it achieved a median scores of 99.7. The number "99.7" should to be "89.13", as show in table 3? The SD value of GetOrganelle is too large than the other three tools but it achieved the highest scores.

Part of "Consistency"

Line 3: As shown in the Fig.6, only two tools achieved R2 not equal 1 by rerunning assemblies and comparison of the scores of two assemblers, however, GetOrganelle defined as the only tool that succeed in obtaining similar score for all assemblers, this conclusion is not rigorous.

*We addressed the more specific issues within the manuscript.*

**Second round of review**

**Reviewer 1**

**Are you able to assess all statistics in the manuscript, including the appropriateness of statistical tests used?**

No, I do not feel adequately qualified to assess the statistics.

**Comments to author:**

Please consider the following questions:
- Are the methods appropriate to the aims of the study, are they well described, and are necessary controls included? If not, please specify what is required.
YES
- Are the conclusions adequately supported by the data shown? If not, please explain
YES
- Are sufficient details provided to allow replication and comparison with related analyses that may have been performed? If not, please specify what is required.
YES
- Does the work represent a significant advance over previously published studies?
YES
- Is the paper of broad interest to others in the field, or of outstanding interest to a broad audience of biologists?
YES

This version is much improved with testing of more than hundred real data sets of unpublished chloroplast genomes.  Authors also provide new chloroplast genome sequences for many species and asses inter/intra-individual differences.  Among genomes investigated, almost 50% had no reference sequence in the chloroplast genome data base shows important application of these tools.  Authors also point out strengths and weaknesses of each tool. Figure 7 shows lack of phylogenetic bias, facilitating broader application of these tools.  Most of the concerns raised in previous review has been addressed.  Only comment I have is to follow up on author's statement in the introduction on biotechnology applications of chloroplast genomes.  It would be helpful to identify crop chloroplast genomes newly identified in this analysis which had no reference sequence in the database.  This would be helpful in further advancing this field.

**Reviewer 2**

**Are you able to assess all statistics in the manuscript, including the appropriateness of statistical tests used?**

Yes, and I have assessed the statistics in my report.

**Comments to author:**

In this report, the authors compared the performance of different assembly tools of chloroplast genome and present a benchmark of seven chloroplast assembly tools, capable to succeed in more than 60 % of known real data sets. The results show significant differences between the tested assemblers in terms of generating whole chloroplast genome sequences and computational requirements. Further, they  created docker images for each tested tool that are freely available for the scientific community and ensure reproducibility of the analyses. Overall, this is a typical
Bio-informatics research and is in the scope of Genome Biology.
   The major concern for this manuscript is about its novelty since the authors just compared seven chloroplast assembly tools developed by others and not form your own research.
    The another concern is regarding its potential application of this assessment for the chloroplast genome assembly in the future. Before the emergence of the Single molecule real-time sequencing (SMRT) and Oxford Nanopore sequencing technology, the Illumina sequencing platform is the mainstream DNA sequencing method and it will generate small reads (100-500 bp), which means there will have a lots of " gaps" between the short reads and they will dramatically hinder the assembly of complete chloroplast genome. In 2012, my lab also try to assemble a chloroplast genome with the genome size 160 Kb by using the Illumina sequencing platform. Unfortunately, there have more than 200 gaps in the draft genome and then we spent almost 1 year to fill these gaps by several other strategies (increasing the sequencing depth, PCR amplification and so on) , that makes us exhausted. However, we performed the DNA sequencing for the same plant species by SMRT and Oxford Nanopore recently, only two gaps in the SMRT sample and no any gap from the Oxford Nanopore sample. Because the average reads length of SMRT is around 10 kb and the maximum is 30-40 kb , for the Oxford Nanopore, the average reads length is around 15 kb and the maximum is 300 kb! In recently, there have considerable cases showed that it is quite easy to obtain complete chloroplast genome assembly without specific chloroplast assembly tools because of the significant increase in the read length

**Authors Response**

**Point-by-point responses to the reviewers’ comments:**

In this report, the authors compared the performance of different assembly tools of chloroplast genome and present a benchmark of seven chloroplast assembly tools, capable to succeed in more than 60 % of known real data sets. The results show significant differences between the tested assemblers in terms of generating whole chloroplast genome sequences and computational requirements. Further, they  created docker images for each tested tool that are freely available for the scientific community and ensure reproducibility of the analyses. Overall, this is a typical
Bio-informatics research and is in the scope of Genome Biology.
   The major concern for this manuscript is about its novelty since the authors just compared seven chloroplast assembly tools developed by others and not form your own research.
    The another concern is regarding its potential application of this assessment for the chloroplast genome assembly in the future. Before the emergence of the Single molecule real-time sequencing (SMRT) and Oxford Nanopore sequencing technology, the Illumina sequencing platform is the mainstream DNA sequencing method and it will generate small reads (100-500 bp), which means there will have a lots of " gaps" between the short reads and they will dramatically hinder the assembly of complete chloroplast genome. In 2012, my lab also try to assemble a chloroplast genome with the genome size 160 Kb by using the Illumina sequencing platform. Unfortunately, there have more than 200 gaps in the draft genome and then we spent almost 1 year to fill these gaps by several other strategies (increasing the sequencing depth, PCR amplification and so on) , that makes us exhausted. However, we performed the DNA sequencing for the same plant species by SMRT and Oxford Nanopore recently, only two gaps in the SMRT sample and no any gap from the Oxford Nanopore sample. Because the average reads length of SMRT is around 10 kb and the maximum is 30-40 kb , for the Oxford Nanopore, the average reads length is around 15 kb and the maximum is 300 kb! In recently, there have considerable cases showed that it is quite easy to obtain complete chloroplast genome assembly without specific chloroplast assembly tools because of the significant increase in the read length

*Please excuse the delay of our resubmission due to my parental leave and holiday season. I hope, we included all information requested by your decision letter. In more detail, we used your suggested title and abstract. Additionally, we examined the species with novel chloroplasts and identified 7 sequences (out of 20) used as ornamental plant, in folk medicine, or as crop plant. All requested changes for referencing tables and figures in the additional file 1 are included.*
